# AKAP95 regulates splicing through scaffolding RNAs and RNA processing factors

**DOI:** 10.1038/ncomms13347

**Published:** 2016-11-08

**Authors:** Jing Hu, Alireza Khodadadi-Jamayran, Miaowei Mao, Kushani Shah, Zhenhua Yang, Md Talat Nasim, Zefeng Wang, Hao Jiang

**Affiliations:** 1 Department of Biochemistry and Molecular Genetics, UAB Stem Cell Institute, University of Alabama at Birmingham School of Medicine, Birmingham, Alabama 35294, USA; 2 Lineberger Comprehensive Cancer Center, Department of Pharmacology, University of North Carolina, Chapel Hill, North Carolina 27599, USA; 3 University of Bradford School of Pharmacy, Bradford BD7 1DP, UK

## Abstract

Alternative splicing of pre-mRNAs significantly contributes to the complexity of gene expression in higher organisms, but the regulation of the splice site selection remains incompletely understood. We have previously demonstrated that a chromatin-associated protein, AKAP95, has a remarkable activity in enhancing chromatin transcription. In this study, we show that AKAP95 interacts with many factors involved in transcription and RNA processing, including selective groups of hnRNP proteins, through its N-terminal region, and directly regulates pre-mRNA splicing. AKAP95 binds preferentially to proximal intronic regions on pre-mRNAs in human transcriptome, and this binding requires its zinc-finger domains. By selectively coordinating with hnRNP H/F and U proteins, AKAP95 appears to mainly promote the inclusion of many exons in the genome. AKAP95 also directly interacts with itself. Taken together, our results establish AKAP95 as a mostly positive regulator of pre-mRNA splicing and a possible integrator of transcription and splicing regulation.

## Abstract (web summary)

The chromatin-associated protein AKAP95 is known for its chromatin-related functions including enhancing transcription. Here the authors show that AKAP95 interacts with the splicing regulatory factors as well as RNAs to regulate the inclusion of exons and pre-mRNA splicing.

## Graphical abstract

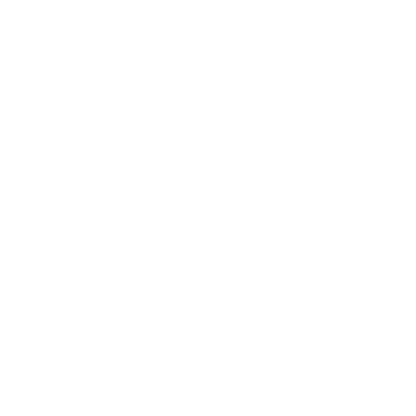

Nascent messenger RNA (mRNA) precursors (pre-mRNAs) in eukaryotic cells are subject to extensive processing, including splicing to remove introns, to generate mature mRNAs. These processing steps are accomplished by coordinated action of many RNA-interacting proteins and RNAs. Alternative splicing (AS) of pre-mRNA is a major source of proteomic diversity in metazoans, as over 95% of human multi-exon pre-mRNAs are processed into two or more mRNA isoforms^1^,^2^, and the frequency of AS correlates with organism complexity along evolution^3^,^4^. AS regulation also plays crucial roles in diverse biological processes^5^, and disruption of AS control can result in human diseases^6^.

The core splicing signals include the 5′ splice site, the 3′ splice site and the branch point sequence, and are primarily recognized by the basal splicing machinery – the spliceosome. In addition to the core splicing signals, numerous *cis*-regulatory elements are present in exons and introns of pre-mRNAs to serve as splicing enhancers or silencers^7^. The functions of the *cis*-elements are mediated by a number of *trans*-acting splicing factors, including SR and SR-related proteins, heterogeneous nuclear ribonucleoproteins (hnRNPs), and other tissue-specific RNA-binding proteins^8^. These factors activate or suppress either splice site recognition or spliceosome assembly via various mechanisms^9^, often in a context-dependent manner^10^.

hnRNP proteins, including at least 20 proteins named hnRNP A1 through hnRNP U, are a structurally diverse group of RNA-binding proteins that participate in many aspects of RNA processing including constitutive and alternative splice regulation^11^. Although earlier studies largely point to a repressive role of hnRNPs in splicing, it is now clear that they both promote and repress splicing^11^,^12^. Some hnRNPs, most notably hnRNP F, H1 and U, have a higher tendency to promote exon inclusion than exclusion^12^. Following their direct binding to pre-mRNAs, hnRNPs can control splice site selection by occluding the binding of splice-regulatory factors to splice sites or splice enhancers and by hindering or facilitating the communication between splice sites. For example, hnRNP A/B and hnRNP F/H have been shown to engage in homotypic or heterotypic interactions and bring different intronic regions into close proximity, thus ‘looping out' the in-between RNA sequence^13^,^14^. The inclusion of a cassette exon can be either promoted or repressed, depending whether the exon is flanking or within the looped out sequence. A rather similar strategy is apparently adopted by hnRNP C, which may also bring two splice sites into close proximity through self-interaction, and thereby promote or inhibit the inclusion of a cassette exon depending on the exon's location relative to the splice sites brought together by hnRNP C (ref. 15).

In addition to the individual *cis*- and *trans*-acting elements, it is increasingly clear that splicing is dynamically regulated within the integrated network of gene expression including transcription^16^,^17^, as most splicing events occur cotranscriptionally. Several splicing factors have been shown to physically interact with the basal transcriptional machinery, and the rate of transcription elongation can play important roles in regulating the splice site selection^17^. Moreover, chromatin structure can also impact AS through the direct recruitment of splicing factors by certain chromatin modifications or through affecting transcription elongation rate^16^. The detailed mechanisms underlying the coupling of transcription and splicing regulation still require much more investigation.

AKAP95 (AKAP8)^18^ is the only nuclear member of the large family of A-kinase anchoring proteins (AKAPs), which bind to protein kinase A and spatiotemprorally regulate cellular signalling^19^. In addition, AKAP95 is the founding member of the AKAP95 family proteins, which also include HA95 (the mammalian AKAP95 paralog) and ZNF326 and all share the common AKAP95 subtype of the zinc-finger domains (ZFs)^20^. Several different functions have been proposed for AKAP95 (refs 21, 22, 23), including mediation of chromatin condensation^24^,^25^ and recruitment of HDACs to mitotic chromosomes^23^. Our previous work^26^ has demonstrated that AKAP95 is associated with the DPY30 subunit of the SET1/MLL complexes, the major histone H3K4 methyltransferases in mammals, and enhances the methylation activity of the complexes on chromatin substrate *in vitro*. AKAP95 strongly stimulates the expression of a chromatin reporter gene, and this effect requires both the N-terminal region (1–100 residues) and the ZFs. We have also shown that AKAP95 regulates retinoic acid-mediated gene induction in mouse embryonic stem (ES) cells. The exact role of AKAP95 in regulating gene expression, however, remains elusive. To further understand the molecular function of AKAP95 in gene regulation, we took an unbiased proteomic approach to identify proteins associated with AKAP95. Unexpectedly, we discovered that AKAP95 mainly associates with many proteins involved in RNA processing, including hnRNP proteins. This led our further studies to reveal a functional role of AKAP95 in regulating pre-mRNA splicing, especially in promoting exon inclusion in AS, by scaffolding RNAs and RNA processing factors. Our work reveals a new activity of AKAP95, which may represent a novel class of splicing factors that bridge the regulation between transcription and splicing.

## Results

### AKAP95 binds to RNA processing factors at its N-terminus

**Figure 1:**
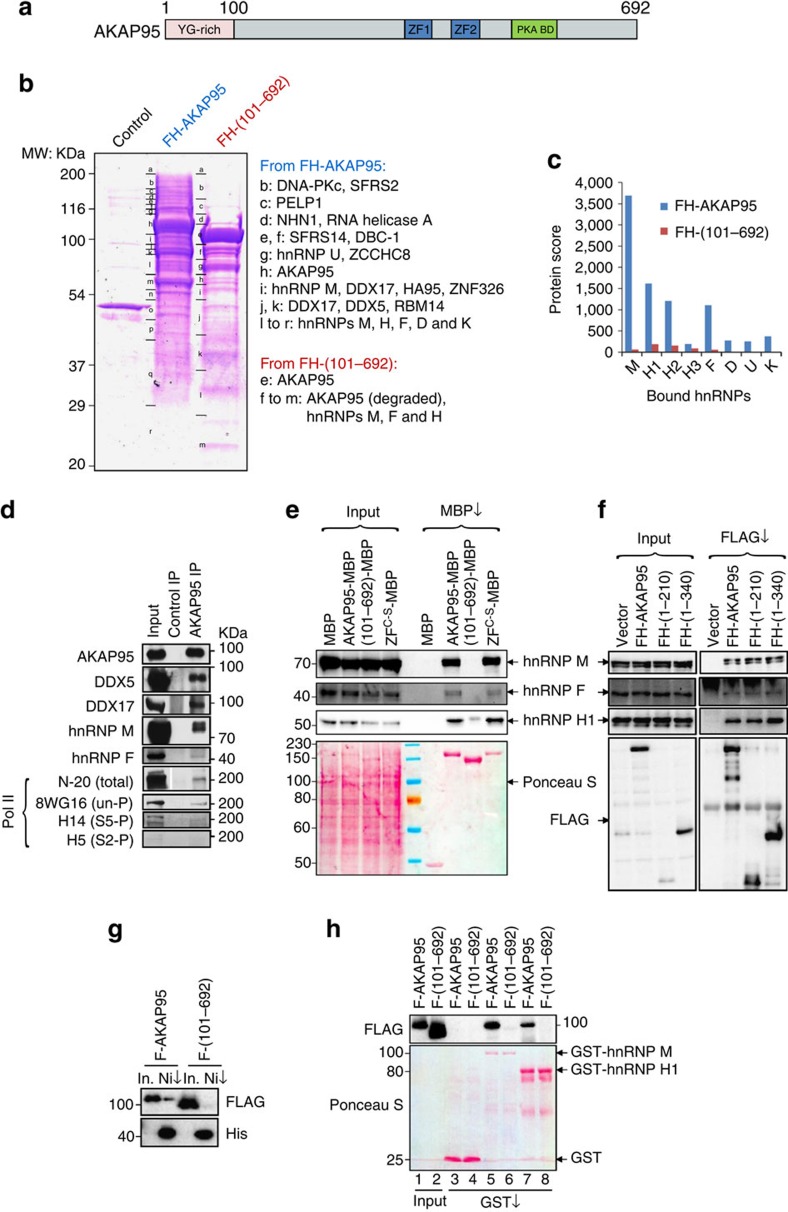
AKAP95 is associated with many proteins involved in transcription and RNA processing at its N-terminus. (**a**) Schematics of domains on AKAP95. See Supplementary Fig. 1a for sequence and annotations. (**b**) Coomassie blue-staining of proteins resolved on SDS–polyacrylamide gel electrophoresis gel following immunoaffinity purification from the parental (control) and 293 cell lines stably expressing FLAG-HA-tagged (FH-) AKAP95 or AKAP95 (101–692). Major proteins identified specifically from the indicated cell line by mass spectrometry are indicated. Lines on the left of the lane indicate positions of gel excision for mass spectrometry. Proteins in bold have protein scores over 200. (**c**) The mass spectrometry protein scores of the identified hnRNP proteins co-immunoprecipitated with FH-AKAP95 or FH-AKAP95 (101–692) are plotted. (**d**) Immunoblot analysis of endogenous AKAP95-associated proteins following immunoprecipitation of HeLa cell nuclear extract by control (normal rabbit IgG) or anti-AKAP95 antibody. (**e**) Immunoblot analysis of immunoprecipitation by amylose beads (MBP↓) of lysates from 293 cells expressing MBP or MBP fused to AKAP95 wild type or mutants as indicated. Antibodies for hnRNPs were used for blotting, and membrane was also stained by ponceau S to show the general loading as well as the comparable precipitation of the MBP or MBP fusion proteins. (**f**) Immunoblot analysis of immunoprecipitation by anti-FLAG antibody-conjugated resin (FLAG↓) of lysates from 293 cells expressing FH-tagged AKAP95 wild type or mutants as indicated. Antibodies for hnRNP proteins or FLAG epitope were used for blotting. (**g**) Direct interaction of AKAP95 with hnRNP F, as shown by immunoblot analysis of the *in vitro* binding assay for purified F-AKAP95 or F-AKAP95 (101–692) with purified His-hnRNP F that remained bound to Ni resin. Inputs (In.) for F-AKAP95 and F-AKAP95 (101–692) were also shown. (**h**) Direct interaction of AKAP95 with hnRNPs M and H1, as shown by immunoblot analysis of the *in vitro* binding assay for purified F-AKAP95 or F-AKAP95 (101–692) with purified GST (lanes 3 and 4), GST-hnRNP M (lanes 5 and 6) and GST-hnRNP H1 (lanes 7 and 8) that remained bound to Glutathione resin. Inputs (lanes 1 and 2) for F-AKAP95 and F-AKAP95 (101–692) were also shown.

Because the N-terminal region (1–100 residues) of AKAP95 is highly enriched with Tyr-Gly (YG) motifs (Fig. 1a and Supplementary Fig. 1a) and required for stimulation of chromatin reporter expression^26^, we reasoned that this region may be associated with factors crucial for gene expression, in addition to DPY30 complexes. We thus sought to identify its binding proteins by immunoaffinity purification of nuclear extracts derived from HEK293 cell lines that stably express FLAG-HA-tagged (FH-) full-length human AKAP95 (FH-AKAP95) or FH-AKAP95 (101–692), followed by mass spectrometric analysis (Fig. 1b and Supplementary Table 1). We found that the major AKAP95-assoicated proteins (mass spectrometric protein score >200) are known to play important roles in many aspects of RNA processing, including hnRNP M, K, H, F, D and U proteins, and DDX5, DDX17, DBC1 and RBM14. Proteins with intermediate scores (between 40 and 200) included EWS, PELP1, NHN1, RNA helicase A, and the other two members of the AKAP95 family, HA95, and ZNF326. Two SR proteins, SFRS2 and SFRS14, were also detected with relatively low scores. We note that the abundant hnRNP A1 was not recovered in the FH-AKAP95 pull-down, suggesting that AKAP95 selectively interacts with hnRNP proteins. In contrast to FH-AKAP95, although FH-AKAP95 (101–692) was expressed at a slightly higher level, its pull-down consisted mainly of AKAP95 (101–692) and its degraded forms (Fig. 1b). None of the other AKAP95-associated proteins, except for hnRNPs M, H and F with much lower scores, was recovered in the pull-down of AKAP95 (101–692; Fig. 1c and Supplementary Table 1), indicating an important role of the N-terminal region (1–100 residues) for binding the RNA processing factors.

The endogenous association of some of these proteins was confirmed by their co-immunoprecipitation with AKAP95 from HeLa cell nuclear extract (Fig. 1d). Although the mass spectrometric analysis did not find RNA polymerase II (Pol II), our immunoblotting assays showed that AKAP95 was associated with Pol II that was unphosphorylated or phosphorylated at serine 5 or 2 of its C-terminal domain, although the association of serine 2-phosphorylated Pol II is less certain due to the overall low signal (Fig. 1d). The association of AKAP95 with these proteins was not mediated by RNAs, as effective treatment of the nuclear extract with RNAse A had little effect on the efficiency of the co-immunoprecipitation (Supplementary Fig. 1b,c).

Consistent with the mass spectrometric analysis, our immunoblotting analysis from separate pull-down assays also demonstrated that AKAP95 (101–692) did not bind efficiently to hnRNPs M, F or H1 (Fig. 1e). However, AKAP95 ZF^C-S^ mutant (with Cys to Ser mutation in both zinc fingers, Supplementary Fig. 1a)^26^ bound as efficiently to these hnRNPs as the wild-type AKAP95 (Fig. 1e). Importantly, the N-terminal region of AKAP95 was sufficient to mediate binding to these hnRNPs, as shown by the efficient co-immunoprecipitation of these proteins by FH-AKAP95 (1–210) and FH-AKAP95 (1–340; Fig. 1f). AKAP95 (1–100) could not be tested due to its poor expression or stability in cells. Moreover, we demonstrated a direct binding of purified hnRNPs F, H1 and M with AKAP95, but not with AKAP95 (101–692; Fig. 1g,h). Furthermore, AKAP95 appeared to bind more strongly with hnRNP M than H1 *in vitro* (Fig. 1g,h), suggesting a differential binding affinity between AKAP95 and hnRNPs. These results indicate that the N-terminal region of AKAP95 critically mediates binding to the RNA processing factors including selective groups of hnRNPs, while its ZFs are dispensable for binding to these factors.

### AKAP95 directly regulates splicing of a minigene pre-mRNA

**Figure 2:**
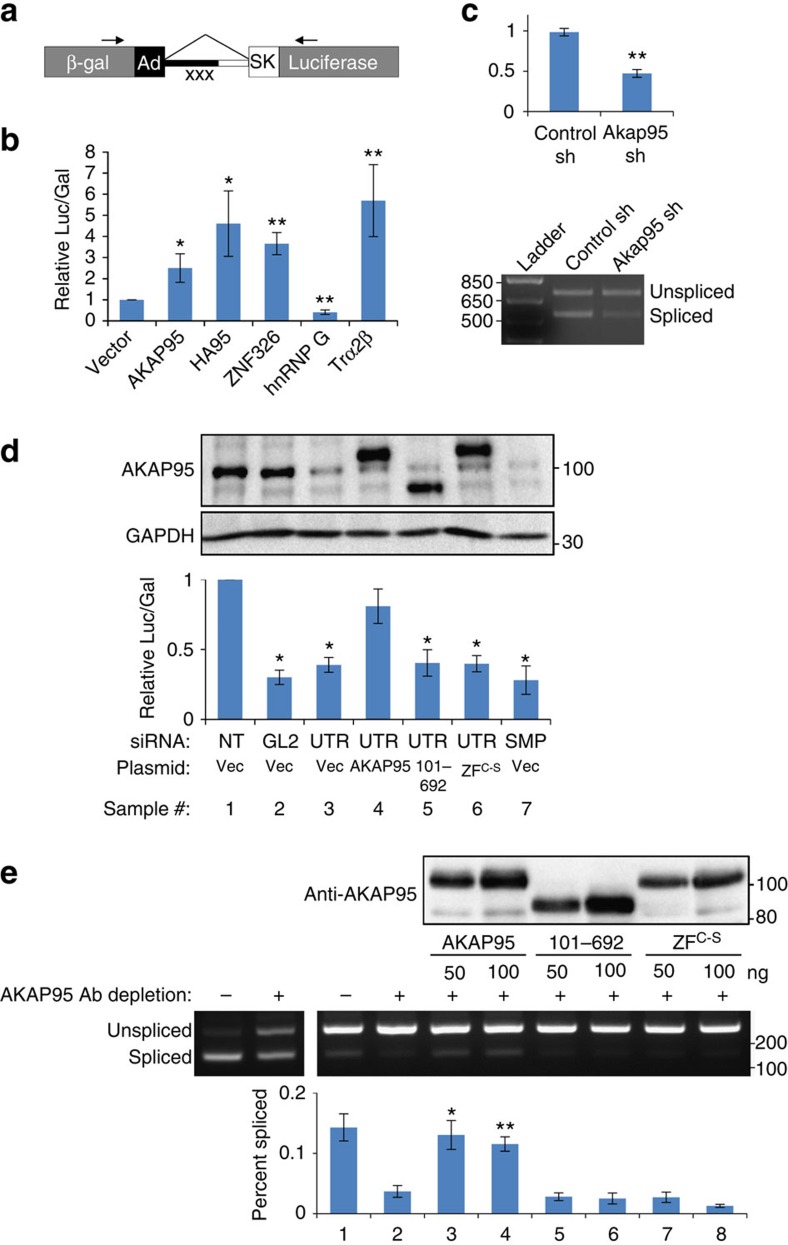
AKAP95 is directly required for efficient minigene splicing. (**a**) Diagram of the minigene splice reporter. ‘xxx' represents three stop codons inserted into the intron. Arrows represent PCR primers used in **c**. (**b**) 293 cells co-transfected with the splice reporter and the indicated plasmids were subject to the double reporter assay. hnRNP G and Trα2β, known to be negative and positive regulators of the minigene splicing, respectively^27^, were included as controls. Average±s.d. from four independent transfections are plotted. *P<0.01, **P<0.001 between vector transfection and the indicated sample. (**c**) The splice reporter was transfected into the NIH3T3 cells after stable infection of control or Akap95 shRNA. Splicing was determined in two ways including the double reporter assay (top) and the PCR-based assay (bottom). Average±s.d. from three biological repeats are plotted. **P<0.001 between the two samples. (**d**) 293 cells co-transfected with indicated siRNAs and plasmids were subject to immunoblotting with indicated antibodies (top) and the double reporter assay (bottom). NT is a non-targeting siRNA. GL2 siRNA targets the luciferase RNA. Average±s.d. from three independent transfections are plotted. *P<0.01 between the indicated sample and either sample 1 or 4. *P*>0.05 between samples 1 and 4. (**e**) *In vitro* splicing assays using two different batches of HeLa nuclear extracts, with batch 1 (left gel) much more concentrated and thus more active than batch 2 (right gel). Batch 2 nuclear extract was from HeLa cells with AKAP95 stably knocked down by shRNA. HeLa nuclear extract was depleted of AKAP95 by antibody when indicated by ‘+'. Two different doses of purified recombinant AKAP95 or its mutants were added as indicated. Immunoblotting (top) indicates the levels of the exogenous wild type or mutated AKAP95. The Percentage of spliced product in the total product (percent spliced) was calculated based on quantification of band intensities, and plotted as average±s.d. from three independent splicing assays (bottom). *P<0.01, **P<0.001 between the indicated sample and any of samples 2, 5–8.

The physical association of many RNA processing factors with AKAP95 suggests a potential function of AKAP95 in RNA processing such as regulation of pre-RNA splicing. We thus set out to test this hypothesis, starting with a minigene splicing system (Fig. 2a) that allows a convenient splicing readout by a double reporter assay^27^. We found that the splicing efficiency of the minigene in human 293 cells was enhanced by overexpression of AKAP95, as well as any of the other two members of the AKAP95 family, HA95, and ZNF326 (Fig. 2b). Conversely, the minigene was spliced less efficiently following a short-hairpin RNA (shRNA)-mediated knockdown (KD) of Akap95 in mouse NIH3T3 cells, as demonstrated by both the double reporter and the PCR-based assays (Fig. 2c). Similarly, the splicing efficiency of the minigene was reduced by AKAP95 KD via small-interfering RNAs (siRNAs) either specific for the 3′-untranslated region (3′UTR) of *AKAP95* or with mixed specificity for *AKAP95* (Smart-pool siRNA, SMP) in human 293 cells (Fig. 2d). Importantly, splicing was rescued by wild-type AKAP95 that was expressed near the endogenous level, but not the AKAP95 (101–692) or AKAP95 ZF^C-S^ mutants that were expressed at similar levels as wild type (Fig. 2d). These results show that the efficient minigene splicing in cells requires AKAP95, and both its N-terminal 100 residues and ZFs are important for this regulation.

We next asked if AKAP95 was directly involved in splice regulation in a cell-free splicing reaction. First we showed that our HeLa cell nuclear extract could efficiently splice the minigene pre-mRNA in a manner that was sensitive to salt concentration and heat (Supplementary Fig. 2a), consistent with the established *in vitro* splicing system^28^. The derived PCR products were also sequence-verified to represent unspliced and spliced species. We showed that antibody-mediated AKAP95 depletion markedly reduced the splicing of the minigene transcript (Fig. 2e, left gel). To achieve a cleaner AKAP95 depletion, we used HeLa cells in which AKAP95 had been stably knocked down by shRNA, and also used a modified protocol to derive nuclear extract from these cells at a much less protein concentration. Further AKAP95 depletion by antibody from this nuclear extract reduced the pre-mRNA splicing (Fig. 2e, compare lanes 1 and 2 on the right gel). Importantly, addition of two different doses of purified wild-type AKAP95, but not the AKAP95 (101–692) or AKAP95 ZF^C-S^ mutants (Supplementary Fig. 2b) at similar levels, nearly fully restored splicing in this cell-free system (Fig. 2e). These results indicate that AKAP95 directly regulates the minigene pre-mRNA splicing, and both its N-terminal region and ZFs are required for the direct regulation.

### AKAP95 directly regulates alternative splicing of *FAM126A*

**Figure 3:**
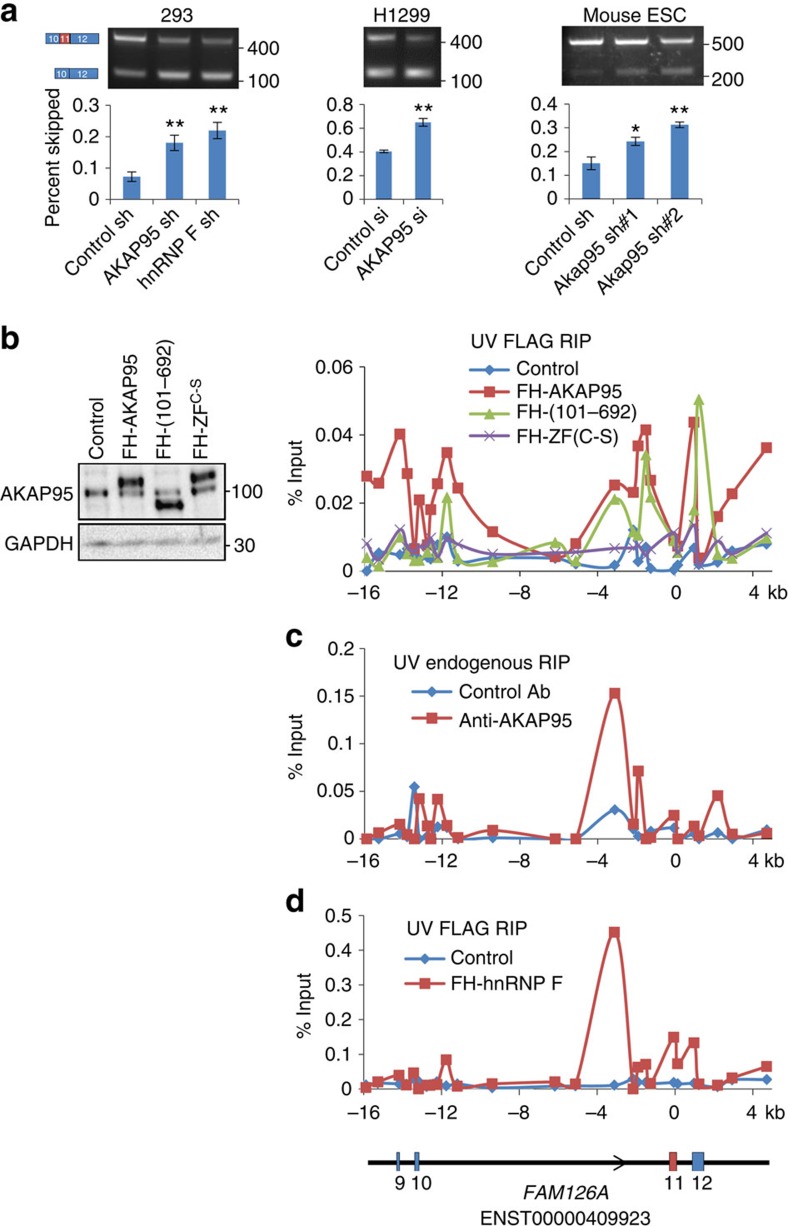
AKAP95 directly regulates AS of *FAM126A*. (**a**) PCR assays for skipping of *FAM126A* exon 11 following AKAP95 KD in human 293 and H1299 cells and also in mouse ES cells using indicated shRNAs (for 293 and ES cells) or siRNAs (for H1299). Scramble shRNA was used as control shRNA, and GL2 siRNA targeting luciferase RNA was used as control siRNA. Following quantification of band intensities by the ImageJ programme, the percentage of the PCR product representing exon 11-skipped transcript in the total transcripts was calculated and shown as ‘percent skipped' on the *y* axis. For all cells, average±s.d. from three biological repeats are plotted. *P<0.01, **P<0.001 between the indicated sample and their corresponding control sample. (**b**) Expression of FH-AKAP95 or the indicated mutants was induced by doxycycline in stable Flp-In T-REx 293 cell lines. The control is the parental Flp-In T-REx 293 cells. The expression levels were shown by western blotting for AKAP95 (left). Anti-FLAG RIP assays were performed in these cell lines after ultraviolet-crosslinking, and followed by qPCR assays with a series of primers (represented by dots on the curves) at the *FAM126A* locus (diagram at the very bottom of this figure). Another biological repeat of RIP assay is shown in Supplementary Fig. 3d. (**c**) RIP assays using control (normal rabbit IgG) or anti-AKAP95 antibody were performed in 293 cells after ultraviolet-crosslinking, and followed by qPCR by the same series of primers as in **b** at the *FAM126A* locus. Two more independent biological repeats are shown in Supplementary Fig. 3e. (**d**) 293T cells were transfected with empty vector (control) or the FH-hnRNP F plasmid to express sub-endogenous level of FH-hnRNP F using a plasmid dose equivalent to that circled in Supplementary Fig. 3g. Anti-FLAG RIP assays were performed after ultraviolet-crosslinking, and followed by qPCR at *FAM126A*. Two more independent biological repeats are shown in Supplementary Fig. 3f.

We next sought an endogenous target of AKAP95 for splicing regulation. Considering the direct interaction of AKAP95 and hnRNP F, we examined the effect of AKAP95 KD on splicing events that were established to be regulated by hnRNP F, including the AS of exon 11 of the *FAM126A* transcript (FAM126A-006 ENST00000409923)^12^. Comparable to the effect of hnRNP F KD (Fig. 3a), AKAP95 KD mediated by specific shRNA or siRNA in different human cell lines consistently resulted in increased skipping (reduced inclusion) of exon 11 in *FAM126A* transcript (Fig. 3a). Importantly, AKAP95 KD did not affect the expression of hnRNPs at the RNA or protein levels (Supplementary Fig. 3a,b). Moreover, Akap95 KD mediated by two different shRNAs in mouse ES cells also impaired inclusion of exon 10 (the corresponding exon of the human exon 11) in mouse *Fam126a* (Fig. 3a, right, and Supplementary Fig. 3a,b), suggesting a conserved role of AKAP95 in regulating the AS of *FAM126A*.

To determine if AKAP95 directly regulates *FAM126A* splicing, we performed RNA immunoprecipitation (RIP) assays following either formaldehyde- or ultraviolet-mediated crosslinking of cells. Formaldehyde tends to provide higher crosslinking efficiency than ultraviolet, but it also crosslinks proteins with interacting proteins, while ultraviolet-crosslinking is only effective for direct RNA-protein contact. To achieve robust RIP signals in our earlier RIP assays, we induced the expression of FH-AKAP95, FH-AKAP95 (101–692) or FH-AKAP95 ZF^C-S^ to high levels in 293 cells (Supplementary Fig. 3c), and performed formaldehyde crosslinking. Our RIP assays included extensive DNAse treatment followed by reverse transcription of purified RNAs into DNAs, which were used in quantitative PCR (qPCR) reactions. Moreover, the qPCR signals were abolished when the reverse transcriptase SS III was omitted from the reaction (Supplementary Fig. 3c), indicating that the RIP signals were indeed from RNAs.

Our RIP-qPCR assays demonstrated that FH-AKAP95 bound to three major sites in the region around the AKAP95-regulated exon 11 of *FAM126A* (Supplementary Fig. 3c). The first two sites are at the intronic regions near the junctions to exons 10 and 11; and the last one is at exon 12, which contains the 3′UTRs. These RIP signals were specific to FH-AKAP95, as they were largely absent from the negative control cells (Supplementary Fig. 3c). RNA binding of FH-AKAP95 (101–692) was largely similar (or modestly reduced in certain regions) to that of FH-AKAP95, but FH-AKAP95 ZF^C-S^ was defective in binding of RNA at all three major sites, despite their similar expression levels (Supplementary Fig. 3c). These results indicate that the ZFs, but not the N-terminal 1–100 residues, are critical for AKAP95 binding to *FAM126A* pre-mRNA.

We then performed RIP assays following ultraviolet crosslinking to detect direct RNA-protein binding under conditions that allowed comparable expression of FH-AKAP95 and the mutants to the endogenous level (Fig. 3b and Supplementary Fig. 3d). Although the overall signal intensity was lower, the RIP results under these conditions (Fig. 3b and Supplementary Fig. 3d) are highly similar to our previous results on formaldehyde-crosslinked RIP assays for overexpressed FH-AKAP95 (Supplementary Fig. 3c). Moreover, we also performed RIP assays for endogenous AKAP95 following ultraviolet-crosslinking (Fig. 3c and Supplementary Fig. 3e), and the profile highly resembles that following formaldehyde-crosslinking, further supporting that AKAP95 directly binds to *FAM126A* pre-mRNA. We also showed that Akap95 in mouse ES cells bound to largely similar regions near the Akap95-regulated exon 10 of *Fam126a* (Supplementary Fig. 3h), indicating a conserved AKAP95 binding pattern for regulating exon inclusion in *FAM126A*.

We also determined the direct binding of hnRNP F to the *FAM126A* pre-mRNA following ultraviolet-crosslink of 293 cells expressing sub-endogenous level of FH-hnRNP F (Supplementary Fig. 3g), as our anti-hnRNP F antibody did not work robustly in RIP for the endogenous hnRNP F. Consistent with the direct binding between hnRNP F and AKAP95 (Fig. 1g), FH-hnRNP F was also found to directly bind to the same intronic sites as AKAP95 (Fig. 3d and Supplementary Fig. 3f). These results thus establish a direct and joint regulation of *FAM126A* exon 11 inclusion by the interacting AKAP95 and hnRNP F proteins.

### AKAP95 binds to intronic regions of cellular pre-mRNAs

**Figure 4:**
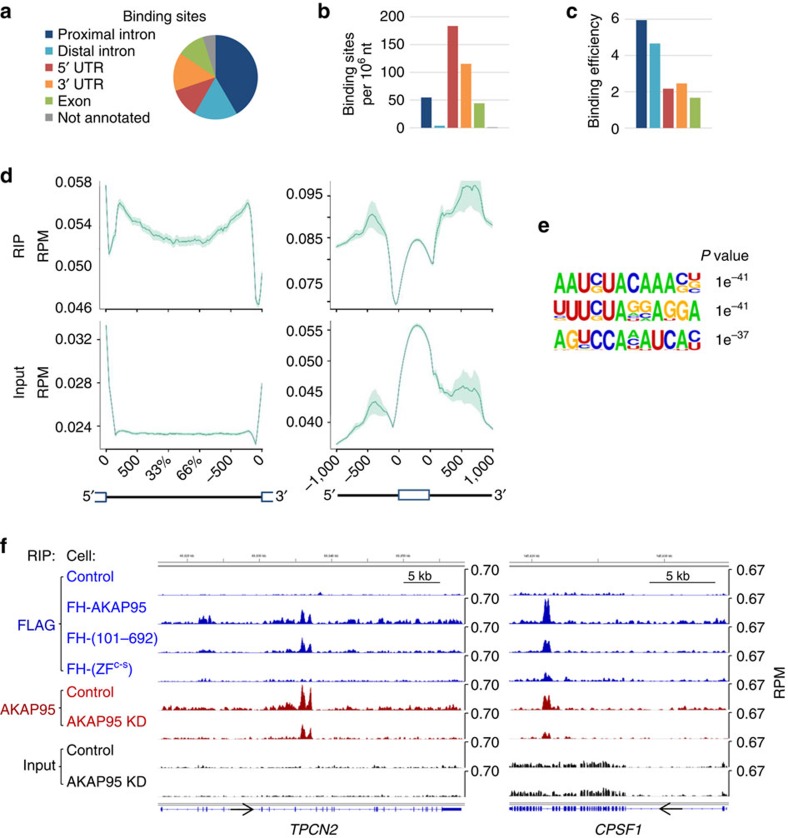
AKAP95 preferentially binds to introns of cellular pre-mRNAs. (**a**) Distribution of the numbers of endogenous AKAP95 binding sites within different categories of genic regions, as calculated from MACS peak counts. The colour legend applies to **a**–**c**. Results in **a**–**e** are from anti-AKAP95 RIP assays in 293 cells. (**b**) Number of AKAP95 binding sites in each 1 million nucleotides of different genic regions. (**c**) AKAP95 binding efficiencies to different categories of genic regions, as calculated as the ratio of the anti-AKAP95 RIP coverage over the input RNA coverage in the corresponding genic regions. (**d**) Profiling plots of AKAP95 binding across a composite intron (left) or exon (right). Both the endogenous AKAP95 binding profile (RIP, top) and the corresponding input RNA profile (Input, bottom) are shown. For the diagram at the bottom, solid line, intron; open box, exon. UTR-containing exons are excluded from the analysis on the right. (**e**) The most enriched sequence motifs bound by AKAP95 in the transcriptome. (**f**) Representative RIP profiles. In blue are the Anti-FLAG RIP-seq profiles from parental 293 cells (control) or 293 cells stably expressing the FH-tagged AKAP95 wild type or mutants as indicated. In red are the anti-AKAP95 RIP-seq profiles from parental 293 cells (control) or 293 cells stably expressing AKAP95 microRNAs (miR#12, shown as AKAP95 KD). In black are the sequencing profiles of total input RNAs from the corresponding cells for the anti-AKAP95 RIP-seq. All profiles have the same *y* axis scale for each gene so that they are directly comparable. Arrows on the gene diagrams indicate the direction of the gene transcription.

We then determined binding of the endogenous AKAP95 to the human transcriptome by deep sequencing analyses of the RNAs enriched from the anti-AKAP95 RIP assays in 293 cells following formaldehyde crosslinking due to its robustness. Extensive binding sites of the endogenous AKAP95 were identified by the RIP-seq analyses, and the specificity of the AKAP95 RIP signals was supported by the diminished RIP signals following AKAP95 KD (Fig. 4f and Supplementary Fig. 3b). Analyses of the RIP-seq results show that AKAP95 binds primarily to intronic regions (Fig. 4a,f, and Supplementary Fig. 4e,f), especially in close proximity (within 500 nt) to exon–intron junctions (Fig. 4a,d). Although AKAP95 also binds to the 5′ and 3′UTRs as well as exons (Fig. 4a,b), the binding at these regions is less efficient than the intronic regions (Fig. 4c), as these regions are much more abundant than the intronic regions in the total RNA input. AKAP95 tends to bind to AU-rich motifs, although several different motifs are associated with AKAP95 (Fig. 4e).

To understand the domain contribution to the transcriptome binding, we also determined the RNA-binding profiles of the stably expressed wild type and mutated AKAP95. The RNA-binding profile of the overexpressed wild-type FH-AKAP95 is overall similar to that of the endogenous AKAP95 (Fig. 4f and Supplementary Fig. 4a–c,e,f), indicating that its overexpression did not grossly change the transcriptome binding. The RIP-seq results also confirmed AKAP95 binding to the intronic regions near the regulated exon 11 of *FAM126A* (Supplementary Fig. 4e). In comparison to the wild type, AKAP95 (101–692) mutant showed similar binding pattern at many transcripts, with either unaffected or modestly reduced signal levels, but the AKAP95 ZF^C-S^ mutant showed markedly reduced binding to the transcripts (Fig. 4f and Supplementary Fig. 4e,f). While these results suggest that the N-terminal region contributes to the RNA binding of AKAP95, they clearly indicate a critical role of the zinc fingers of AKAP95 in transcriptome binding.

We also performed RIP assays for both the overexpressed wild type or mutant AKAP95 and endogenous AKAP95 following ultraviolet-crosslinking of cells, and determined their binding by qPCR at several sites that were shown to be bound by AKAP95 at high levels based on the formaldehyde-crosslinked RIP-seq results. Our results from the ultraviolet- and formaldehyde-crosslinking methods are highly similar for most of these sites, although ultraviolet-crosslinking generated overall lower signal levels (Supplementary Fig. 4d). These results indicate that our RIP assays reflect mostly direct binding of AKAP95 with pre-mRNAs, although indirect binding may also contribute to the RIP signals.

### AKAP95 mainly promotes inclusion of exons globally

**Figure 5:**
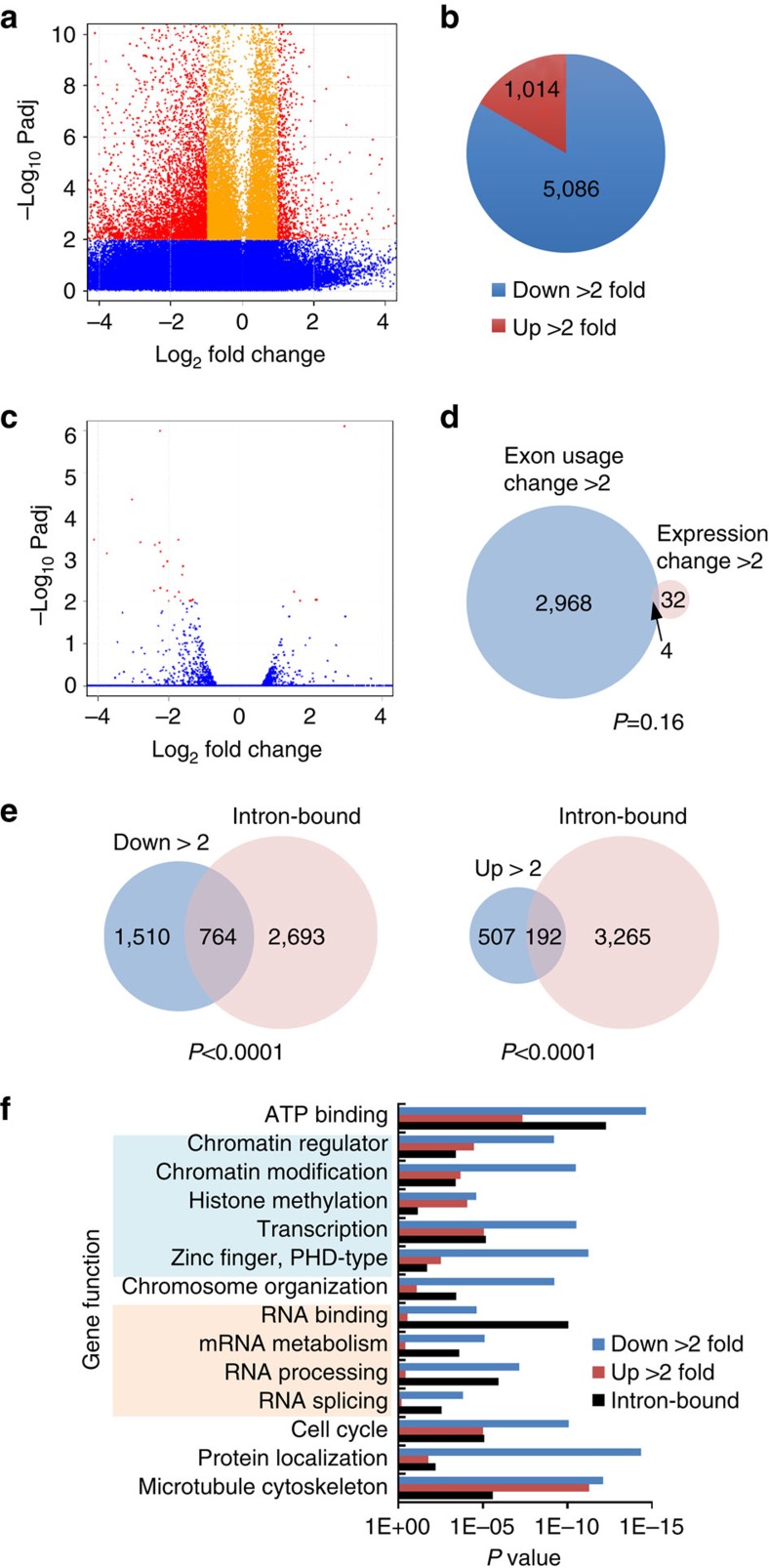
AKAP95 functions to primarily promote exon inclusion. (**a**) Volcano plot for exons whose normalized usage was significantly affected (false discovery rate or Padj<0.01 for red and yellow, red for fold change over 2) or not (Padj>0.01, blue) in 293 cells. Fold change is the ratio of the normalized exon level in AKAP95 KD over that in control cells. See Supplementary Data 1 for the gene list. (**b**) Pie chart showing the number of exons whose normalized usage was significantly (Padj<0.01) reduced (blue) or enhanced (red) over twofold on AKAP95 KD in 293 cells. (**c**) Volcano plot for genes whose expression was significantly affected (Padj<0.01 for red and yellow, red for fold change over 2) or not (Padj>0.01, blue) in 293 cells. Fold change is the ratio of the normalized exon level in the AKAP95 KD over that in the control cells. (**d**) Venn diagram showing an insignificant overlap between genes that contain exons significantly affected (reduced and enhanced) over twofold and genes whose expression was significantly changed on AKAP95 KD in 293 cells. The two-tailed *P* value was calculated by *χ*^2^ test with Yates' correction. (**e**) Venn diagram showing significant overlap between genes that contain exons significantly (Padj<0.01) reduced (left) or enhanced (right) over twofold on AKAP95 KD and genes whose transcripts were bound by AKAP95 at the intronic regions in 293 cells. The two-tailed *P* value was calculated by *χ*^2^ test with Yates' correction. (**f**) Gene ontology analysis of functional and physical target genes of AKAP95. Blue and red bars represent genes containing significantly (Padj<0.01) affected exons over twofold on AKAP95 KD in 293 cells, based on 2,189 DAVID IDs with down-regulated exons and 657 DAVID IDs with up-regulated exons. Black bars represent genes whose transcripts were bound by AKAP95 at the intronic regions, based on 1381 DAVID IDs corresponding to 2,000 intron-bound MACS peaks (with the highest peak scores) from the anti-AKAP95 RIP assays in 293 cells. Gene functions in light blue shade are for chromatin and transcription regulation, and gene functions in pink shade are for RNA binding and processing.

To assess the global impact of AKAP95 on AS, we depleted AKAP95 in human 293 cells and mouse ES cells, and performed RNA-seq followed by DEXseq analysis^29^ to identify the differential exon usage in the cellular mRNAs. DEXseq analysis offers reliable control of false discoveries by taking biological variation into account^29^. AKAP95 KD resulted in much more events of exon usage decrease than increase in both 293 (Fig. 5a,b and Supplementary Data 1) and ES cells (Supplementary Fig. 5a,b and Supplementary Data 1), suggesting that AKAP95 regulates AS globally with a preference to promote exon inclusion. We validated some of the differential exon usage to be *bona fide* exon inclusion or skipping events by PCR-based assays (Supplementary Fig. 5d).

The effects on exon usage reduction are not merely a technical bias due to the reduced expression of some genes, because (1) DEXseq normalizes each exon signal level against the total exon signal levels in the gene^29^, (2) the vast majority of the genes with significantly affected exon usage were not significantly affected in expression by AKAP95 KD in 293 cells (Fig. 5c,d), (3) our PCR data confirms the effects on the skipping or inclusion of specific exons instead of the expression of genes (Supplementary Fig. 5d) and (4) while the expression of similar numbers of genes was up- or down-regulated following Akap95 KD in the mouse ES cells (Supplementary Fig. 5c), the usage of much more exons was increased than decreased (Supplementary Fig. 5a,b).

To determine if the alternative exon usage was a direct effect of AKAP95 activity, we compared the AKAP95 physical targets (based on AKAP95 RIP-seq) with its functional targets (based on mRNA-seq). Genes with AKAP95 bound at its introns were significantly overlapped with genes that showed significant change (either decrease or increase) in exon usage on AKAP95 KD (Fig. 5e), suggesting that many of the differential exon usage events were a direct effect of AKAP95 activity at the RNAs.

To identify biological pathways that the AKAP95 targets are involved in, we performed gene ontology analysis for both the functional and physical targets of AKAP95. Our results reveal a significant enrichment of chromatin/transcription regulators and RNA processing factors in the genes that showed differential exon usages on AKAP95 KD in 293 cells (Fig. 5f) and in mouse ES cells (Supplementary Fig. 5e), as well as in the genes with AKAP95 bound at introns (Fig. 5f, black bars). These results suggest that AKAP95 can potentially regulate global gene expression through direct and indirect control of chromatin, transcription and RNA processing (Supplementary Fig. 5f).

### AKAP95 and selected hnRNPs co-regulate alternative splicing

**Figure 6:**
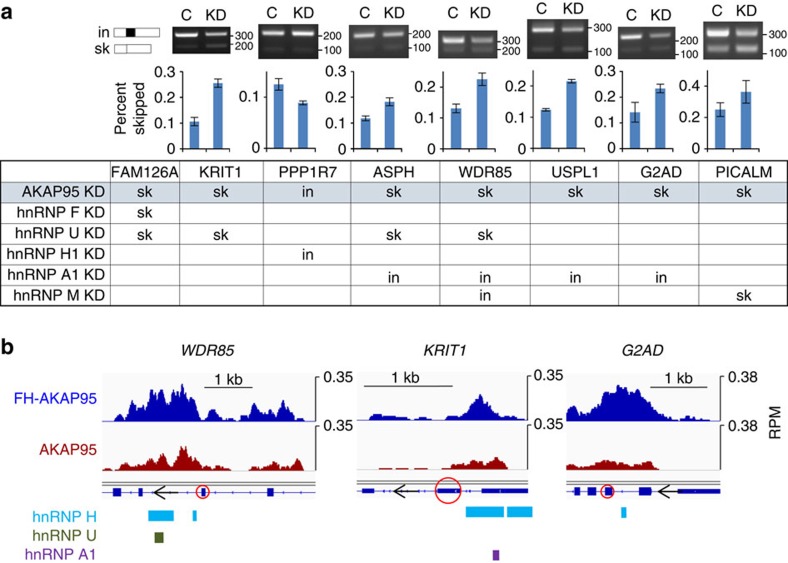
AKAP95 selectively coordinates with hnRNPs to co-regulate AS. (**a**) Effects of AKAP95 KD in 293 cells on AS of exons that were previously shown to be more skipped (sk) or more included (in) following depletion of indicated hnRNPs in the table^12^. Scramble shRNA-expression cells were used as control (‘C'), and AKAP95 shRNA-expressing cells were the KD. ‘Percent skipped' on the *y* axis was calculated as described in Fig. 3a. Average±s.d. from biological duplicates are plotted. *P*<0.05 between control and KD for all these genes except *G2AD* and *PICALM*. (**b**) RNA-binding profiles of exogenous and endogenous AKAP95 (blue and red, respectively) at RNA regions flanking the regulated exons (marked by red circle) in **a**. Shown at the bottom are binding regions of hnRNP proteins taken from http://rnabind.ucsd.edu/. Binding profiles of the other genes in **a** are shown in Supplementary Fig. 6a.

To further understand the functional relationship of AKAP95 with different hnRNP proteins, we also examined the effects of AKAP95 KD on exon inclusions previously shown to be affected by depletion of various hnRNPs^12^. We found that, while AKAP95 KD did not affect the expression of hnRNPs (Supplementary Fig. 3a,b), it affected the exon usage in the same direction as the depletion of hnRNP F, U and H1, but not A1 (Fig. 6a). This is consistent with the physical interaction of AKAP95 with hnRNPs H/F and U, but not A1 (Fig. 1b and Supplementary Table 1). Importantly, we show that AKAP95 binds to the introns immediately flanking the regulated exons (Fig. 6b and Supplementary Fig. 6a), and the binding regions often overlap with those bound by hnRNPs H/F and U. Interestingly, although hnRNP A1 and AKAP95 also have overlapping binding regions in immediately flanking introns of certain genes (Supplementary Fig. 6a), these proteins often have different effects on the inclusion of the nearby exons (Fig. 6a). These results further strengthen our model for a direct control of exon inclusion by AKAP95 through selective coordination with hnRNPs.

### AKAP95 interacts with itself

**Figure 7:**
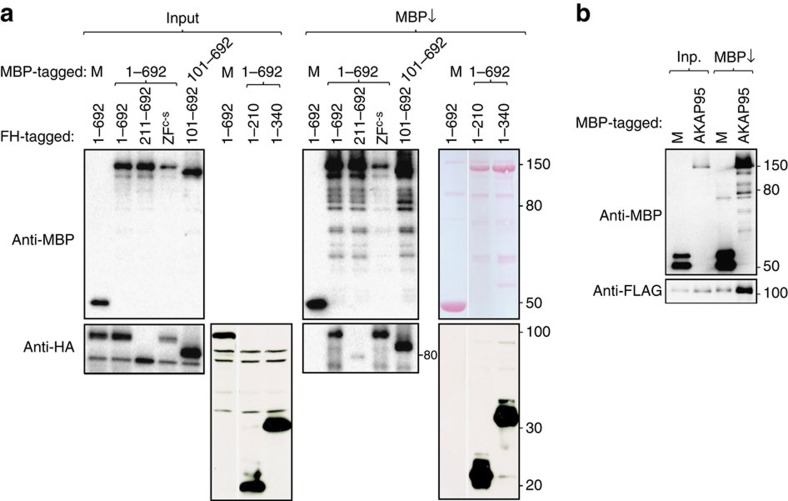
AKAP95 interacts with itself. (**a**) AKAP95 self-interacts through its N-terminal region. 293T cells were co-transfected with MBP-tagged AKAP95 or AKAP95 (101–692) and FH-tagged AKAP95 or mutants as indicated. Unfused MBP (‘M') was used as control. MBP and its fusion proteins were precipitated by amylose resin (MBP↓), and the input and bound proteins were detected by immunoblotting. (**b**) Purified MBP or AKAP95-MBP fusion protein bound with amylose resin (MBP↓) was incubated with purified FLAG-AKAP95 in the binding assay *in vitro*, and the input and resin-bound proteins were detected by immunoblotting.

A loop-out model of action has been proposed for splice regulation by hnRNP A/B and hnRNP F/H proteins through interacting with itself or a different hnRNP protein^13^,^14^. We sought to assess if AKAP95 might act in a similar manner. We found that AKAP95 efficiently interacts with itself, as the FH-AKAP95 could be co-precipitated by the co-transfected AKAP95 fused to maltose-binding protein (MBP), but not by MBP alone (Fig. 7a). We also found that AKAP95 directly interacts with itself, as purified AKAP95-MBP, but not MBP, could efficiently bind to purified F-AKAP95 (Fig. 7b). While AKAP95 (101–692) and AKAP95 ZF^C-S^ were able to bind to AKAP95, AKAP95 (211–692) had a markedly reduced binding capacity with AKAP95. Moreover, AKAP95 (1–210) was sufficient in mediating AKAP95 binding (Fig. 7a). Therefore, the N-terminal region 1–210, especially 101–210, probably plays an important role in AKAP95 self-interaction. These data provide the possibility that, by binding to each end of the RNA intronic sequences and self-interaction, AKAP95 may help bring the two ends of an intron into proximity and thus facilitate the splice site communication and intron definition (Supplementary Fig. 6b).

## Discussion

Although pre-mRNA splicing is a major and key step in the genetic information flow, only a handful of splicing regulators have been shown to be responsible for controlling the large number of AS events in cells^8^. Recent studies^20^,^30^ have identified hundreds of RNA-binding proteins that may potentially regulate many aspects of RNA biology including splicing, but the functional roles of many of them have not been characterized in RNA metabolism. Among these identified RNA-binding proteins are all three members of the AKAP95 family that share the common ZFs. In addition to a role in transcriptional co-activation shown in our previous work^26^, several lines of functional evidence from this study (summarized as follows) collectively support a primarily positive role of AKAP95 in pre-mRNA splice regulation. (1) AKAP95 overexpression enhances splicing of a minigene reporter, and its depletion reduces splicing of the minigene. (2) AKAP95 is required for efficient splicing of a pre-transcribed RNA *in vitro*. (3) Depletion of AKAP95 in both human 293 cells and mouse ES cells primarily result in reduced exon inclusion events. The functional evidence is well in line with our biochemical results that AKAP95 binds to many RNA processing factors including selective groups of hnRNPs, and also directly to pre-mRNAs with a strong preference for introns. The predominant location of AKAP95 in the proximal intronic regions, which are enriched with many intronic splicing regulatory elements^31^,^32^,^33^, is consistent with its role in regulating splice site selection.

We speculate that AKAP95 might facilitate the splice site communication through both RNA-binding and protein–protein interaction (Supplementary Fig. 6b), similar to the proposed mechanisms for hnRNP A1 and H/F^13^,^14^. Such interactions may involve AKAP95 with selective hnRNPs and/or with itself. This model is supported by the functional and biochemical data on at least a few examined target transcripts (for example, *FAM126A* and *WDR85*) of AKAP95 and hnRNP H/F, where AKAP95 binds to both ends of an intron next to the regulated exon. The generality of this model towards other AKAP95 targets awaits further studies, including high-resolution mapping of AKAP95-binding sequences and more detailed biochemical dissections.

Our results suggest that the other two members of the AKAP95 family, HA95 and ZNF326, can physically associate with AKAP95 (Fig. 1b and Supplementary Table 1) and also regulate RNA splicing (Fig. 2b), yet AKAP95 clearly has non-redundant roles as reflected by effects on splicing following its depletion. It will be interesting to understand if these AKAP95 family members regulate RNA processing by similar or different mechanisms, and how cells regulate their differential usage and/or function in RNA processing. ZNF326 has been shown to regulate AS through promoting transcriptional elongation rate^34^, and thus reducing the time window for the weaker splice site recognition before being out-competed by the emerging stronger splice sites. Two lines of evidence suggest that AKAP95 probably does not regulate splicing through the identical mechanisms proposed for ZNF326. (1) Our *in vitro* splicing results indicate that AKAP95 is capable of stimulating splicing even when it is uncoupled from transcription. (2) Exon skipping is much preferred over inclusion upon AKAP95 KD, which is opposite to the preferred exon inclusion upon ZNF326 KD (ref. 34). However, our results do not exclude the possibility that AKAP95 may regulate co-transcription splicing in cells, potentially through a kinetic effect of transcription elongation on exon inclusion with differential consequences.

Our previous^26^ and this studies have established a physical and functional connection of AKAP95 with both transcription and pre-RNA splicing. The N-terminal (1–100) and ZFs of AKAP95 are required for its activity in transcription co-activation as well as splice regulation. Importantly, truncation of the N-terminal region (1–100) is unlikely to affect the overall structure integrity of AKAP95, because mutants lacking this or more extensive N-terminal region retain full activities in (i) mediating chromatin condensation^35^, (ii) binding to a reporter promoter on chromatin^26^ and (iii) binding to many regions in the transcriptome. Rather, the N-terminal region is necessary and sufficient in mediating binding to many hnRNP proteins (Fig. 1f). In addition to RNA Pol II, many other AKAP95 N-terminus-associated proteins including PELP1 (refs 36, 37), RNA helicase A (refs 38, 39, 40), EWS (ref. 41), DDX5 and DDX17 (ref. 42) and RBM14 (CoAA; ref. 43) have all been previously shown to be involved in transcription activation. Therefore, the role of the N-terminal region in both transcription co-activation and splice regulation is most likely mediated by its binding to important factors in both processes. In addition to protein interactions, the N-terminal YG-rich region may also contribute to the RNA-binding capacity of AKAP95, as its deletion modestly impairs binding at some pre-mRNA regions. This is consistent with the observation that YG/RG motifs are frequently found in combination with RNA-binding domains in many RNA-binding proteins^20^. On the other hand, the ZFs are critical for AKAP95 to regulate RNA processing by binding to pre-mRNAs, but their role in transcription co-activation remains elusive. It is worth noting that the zinc-finger subtype in AKAP95 is a new RNA-binding domain that was suggested^20^ but has not been previously experimentally demonstrated. The zinc fingers of AKAP95 are important in mediating chromosome condensation^35^, and might directly or indirectly facilitate AKAP95 association with chromatin. As the communication between transcription and splicing goes both way^16^, it also remains possible that AKAP95 may regulate transcription via its direct impact on RNA binding and splicing.

Cross-regulation of RNA-binding proteins and signal amplification in splicing cascades have emerged as general features of splicing networks, and may serve to stabilize splicing patterns within a cellular state or to expand and reinforce the splicing network^44^. AKAP95 directly modulates the processing of many RNAs whose protein products are involved in RNA processing and chromatin or transcriptional regulation (Fig. 5f and Supplementary Fig. 5e). Consistent with the direct hnRNPs-AKAP95 interaction, many hnRNP proteins are also known to affect the splicing of RNAs encoding RNA-processing factors^12^. Therefore, AKAP95 may coordinate with different hnRNPs to create regulatory cascades for gene expression and function (Supplementary Fig. 5f).

In addition to transcription and splice regulation, AKAP95 may also regulate other steps in RNA processing, as it binds to pre-mRNA UTRs and many factors that play multiple roles in RNA processing. Indeed, AKAP95 binds to 3′ UTR of *LDHA* mRNA together with the regulatory subunits of the protein kinase A, and regulates the stability of that mRNA^22^. NHN1 (ZC3H18) and ZCCHC8, which we found in the AKAP95 immuno-precipitate, are also associated with the cap-binding complex^45^, nuclear exosome targeting complex^46^ and pre-catalytic spliceosomal complexes^47^, and are implicated in multiple steps in RNA processing and metabolism.

Mutations in *FAM126A* have been causally linked to hypomyelination and congenital cataract due to the dysregulation of its pre-mRNA splicing and the consequent deficiency of its protein product, hyccin^48^,^49^. Inclusion of *FAM126A* exon 11, which is regulated by AKAP95 and hnRNP F, leads to a C-terminally truncated hyccin protein (419 compared with 521 residues). It is unclear whether and how such truncation affects the protein function and the potential connection to the disease, but it shows how AKAP95 might regulate human pathology via splice regulation. AKAP95 has also been implicated in modulation of head growth and autism^50^, and found to be significantly overexpressed in rectal cancer together with certain cyclin genes^51^. The latter is consistent with the strong enrichment of the GO term ‘cell cycle' in the AKAP95 bound or regulated targets (Fig. 5f and Supplementary Fig. 5e), and also with the finding that hnRNPs (many of which physically and functionally associate with AKAP95) directly target a large number of cancer-associated genes^12^.

## Methods

### Characterization of protein interactions with AKAP95

The FH-AKAP95 and FH-AKAP95 (101–692) cell lines were made by co-transfecting Flp-In-293 cells (f293 cells, Invitrogen) with pOG44 plasmid and either an FH-AKAP95-pcDNA5 or an FH-AKAP95 (101–692)-pcDNA5 plasmid, followed by Hygromycin B selection for stable clones. Nuclear extracts were obtained from these cell lines by a modified Dignam procedure^52^, incubated with anti-FLAG M2 agarose beads (Sigma) in BC300 (50 mM Tris (pH 7.4), 300 mM KCl, 20% glycerol, 0.2 mM EDTA) with 0.1% NP40 at 4 °C for 6 h and extensively washed with BC300, 0.1% NP40 followed by BC100, 0.1% NP40. The bound proteins were eluted with 0.4 mg ml^−1^ FLAG peptide (Sigma) in BC100 with 0.1% NP40, resolved on SDS–polyacrylamide gel electrophoresis and visualized by coomassie staining. The entire gel lanes were sliced into indicated bands and proteins were subjected to matrix-assisted laser desorption/ionization mass spectrometry. Common background proteins (for example, keratins and tubulins) and the proteins found in the pull-down from the extract of control cells were excluded from the list of interacting proteins. Co-immunoprecipitation of endogenous protein association was carried out in HeLa cell nuclear extract in BC300, 0.1% NP40. Co-immunoprecipitation of overexpressed AKAP95 (and its mutants) with endogenous proteins was performed by transfecting the indicated constructs into 293T cells followed by IP with anti-FLAG M2 agarose beads in cell lysates in BC300, 0.1% NP40. For directly interaction of AKAP95 and hnRNP F, F-AKAP95 and F-AKAP95 (101–692) were recombinantly expressed in sf9 cells and purified as described in our previous publication^26^. Briefly, Flag-tagged AKAP95, AKAP95 (101–692) or AKAP95 ZF^C-S^ cDNA was inserted into the pFastBac1 vector (Invitrogen) and baculoviruses were generated according to the Bac-to-Bac Baculovirus Expression System (Invitrogen) protocols. Baculovirus-infected sf9 cells were lysed in BC500 with 0.05% NP40, 1 mM DTT and protease inhibitor cocktail (Roche). The lysate was incubated with anti-FLAG M2 agarose beads (Sigma), and the beads were extensively washed with the lysis buffer followed by further wash in BC100 with 0.05% NP40. Bound proteins were eluted with 0.4 mg ml^−1^ FLAG peptide (Sigma) in BC100 with 0.05% NP40. His-tagged hnRNP F was purified from sf9 cells infected with baculovirus expressing His-hnRNP F (generously provided by Benoit Chabot, University of Sherbrooke, Canada) using Ni-NTA resin (GE healthcare). hnRNPs M and H1 cDNAs were amplified from 293 cell cDNAs and cloned into pGEX-4T-1 vector (GE Healthcare). glutathione S-transferase (GST), GST-hnRNP M and GST-hnRNP H1 were induced to express in BL21 (DE3) *E.coli* by 0.5 mM of isopropyl β-D-thiogalactopyranoside for 5 h at 22 °C, and purified using glutathione sepharose 4B resin (GE Healthcare) without elution. The resin-bound hnRNP proteins were used in the binding assay with purified F-AKAP95 and F-AKAP95 (101–692) proteins in BC200, 0.1%NP40. For direct self-interaction of AKAP95, MBP and AKAP95-MBP fusion proteins were purified from sf9 cells infected with corresponding baculoviruses using amylose resin (New England BioLabs, E8021S) and remained bound to the resin after extensive washing. The resin-bound proteins were used in the binding assay with purified F-AKAP95 protein in BC200, 0.05% NP40.

### Antibodies

Antibodies were obtained commercially. From Santa Cruz Biotechnology: anti-AKAP95 (sc-10766, 1:500); anti-DDX5 (sc-32858, 1:500); anti-hnRNP M (sc-20002, 1:500); hnRNP F (sc-10045, 1:500); anti-His (sc-803, 1:500); and anti-RNA Pol II (sc-899, 1:500). From others: Anti-hnRNP A1 (Novus Biologicals, NB100–672, 1:2,000), anti-Pol II (Covance, MPY-127R for clones 8WG16, H14 and H5, 1:500), anti-hnRNP H (Bethyl Laboratories, A300–511A, 1:2,000); DDX17 (Bethyl Laboratories, A300–509A, 1:2,000); anti-HA (Abcam, ab9110, 1:1,000); anti-GAPDH (Chemicon, MAB374, 1:5,000); anti-FLAG (Sigma, A8592, 1:1,000); anti-FLAG (Sigma, A2220, M2 beads); and anti-MBP (New England BioLabs, E8032S, 1:5,000).

### Splice reporter assay

The double reporter splicing assay was performed on pTN24 reporter plasmid^27^. Briefly, cells were transfected with relevant plasmids or siRNA using Lipofectamine 2000 reagent (Life Technologies), and harvested 48 h after transfection. β-galactosidase and luciferase activities were measured using Dual-Light System (Applied Biosystems).

### *In vitro* splicing assay

*In vitro* splicing assay was performed using published protocols^28^. Briefly, we used primers (5′-CGCGGATCCGTACTCCCTCTCAAAAGC-3′ and 5′-CCGGAATTCCTTCTCCGCCTGAGCCTC-3′) in a PCR reaction to amplify the DNA fragment containing the exon–intron–exon structure from the pTN23 plasmid^27^ (which does not have a T7 promoter), and cloned it into the pBluescript II KS (−) plasmid (which has a T7 promoter) between BamHI and EcoRI sites. This construct was then linearized by EcoRI and used as substrate in the *in vitro* transcription reaction mediated by T7 RNA polymerase (Promega Part# 9PIP207). A standard splicing reaction contained spliced RNA (0.01–0.1 nM), 20% HeLa nuclear extract, 50 and 100 ng recombinant proteins when indicated, 1 mM ATP, 20 mM creatine phosphate, 3.2 mM MgCl_2_, 10 U ribonuclease inhibitor, 1 mM DTT, 60 mM KCl and 12 mM HEPES-KOH (pH 7.9). The reaction volume was 25 μl. Following incubation at 30 °C for 2 h, reactions were proteinase K digested in 200 μl with 10 mM Tris (pH 7.5), 1% SDS, 0.15 mM NaCl, 1 mM EDTA and 0.2 g l^−1^ proteinase K for 1 h at 42 °C, then phenol chloroform extracted and ethanol precipitated before RT-PCR. To deplete AKAP95 from nuclear extract, AKAP95 antibody was first coupled to CNBr-activated sepharose 4B (Sigma, C9142) following the protocol provided in the Product Information, and incubated with the nuclear extract at 4 °C for 4 h. The supernatant was used as the depleted extract.

### AKAP95 knockdown

We used three different strategies to KD AKAP95 including siRNAs, shRNAs and microRNAs. Their sequences are shown in Supplementary Table 2. Indicated cells were transiently transfected with siRNAs and analysed 2–3 days after transfection. Indicated cells were sometimes infected with shRNA-expressing lentiviruses and selected by puromycin. As an alternative method to establish stable AKAP95-KD cell lines, we also used the BLOCK-iT Pol II miR RNAi Expression Vector Kits (Invitrogen) to generate a plasmid expressing two different AKAP95 microRNA (miR 1 and miR 2) sequences chained as miR1-2-1-2 flanked by BamHI and XhoI restriction sites. The entire microRNAs were transferred into pcDNA5/FRT/TO (Invitrogen) between BamHI and XhoI, and were co-transfected with pOG44 (Invitrogen) into Flp-In T-REx 293 cells (Invitrogen). Stable clones were selected by Hygromycin B, and clones with efficient KD (clones #8 and #12) on doxycycline induction (100 ng ml^−1^) were selected for further studies.

### PCR and qPCR

To determine gene expression or splicing efficiency, total RNAs were extracted using the RNeasy kit (Qiagen) and reverse-transcribed using the SuperScript III First-Strand Synthesis System (Invitrogen) with an oligo dT20 primer. For gene expression, qPCR was performed with SYBR Advantage qPCR Premix (Clontech) on a ViiA7 real-time PCR System (Applied Biosystems). Primers used are listed in Supplementary Table 3. For splicing detection, PCR was performed using EmeraldAmp Master Mix (Takara) with 95 °C 2 min, then 32 cycles of 95 °C 20 s, 58 °C 30 s and 68 °C 50 s. PCR products were resolved on agarose gel, and the signal intensities of the bands were quantified by the ImageJ programme.

### RNA immunoprecipitation

Cells were crosslinked with 1.2% formaldehyde for 15 min or irradiated with ultraviolet light at 254 nm, 400 mJ cm^−2^. Cells (∼50 millions) were harvested and lysed in 2 ml radio-immunoprecipitation assay buffer (50 mM Tris-HCl pH 7.4, 1% NP40, 0.5% sodium dexoycholate, 0.1% SDS, 1 mM EDTA, 150 mM NaCl, 1 mM DTT, 50 units ml^−1^ RNase Out). The cell lysates were sonicated at Bioruptor (Diagenode) at ‘high mode' for 60 cycles to shear to 400–800 bp. After centrifugation, the clear lysate were immunoprecipitated at 4 °C overnight. Beads were washed by low salt (0.1% SDS, 1% Triton X-100, 2 mM EDTA, 20 mM Tris-HCl pH 8.1, 150 mM NaCl), high salt (0.1% SDS, 1% Triton X-100, 2 mM EDTA, 20 mM Tris-HCl pH 8.1, 500 mM NaCl), LiCl immune (0.25 M LiCl, 1% NP40, 1% deoxycholic acid, 1 mM EDTA, 10 mM Tris-HCl pH 8.1), and TE buffer (1 mM EDTA, 10 mM Tris-HCl pH 8.0). After proteinase K digestion and turbo DNase I (4 U per sample) treatment, the RNA was recovered by acidic phenol/chloroform extraction and ethanol precipitation.

### RNA-Seq

RNA-sequencing was performed on the Illumina HiSeq2500 using the sequencing reagents and flow cells providing up to 300 Gb of sequence information per flow cell. Briefly, the quality of the total RNA was assessed using the Agilent 2100 Bioanalyzer. For mRNA-seq, 2 rounds of polyA+ selection was performed. For RIP-seq including the input RNA-seq, no polyA+ or other selection was performed, followed by conversion to cDNAs. We used the stranded mRNA library generation kits per manufacturer's instructions (Agilent, Santa Clara, CA). Library construction consists of random fragmentation of the RNA, followed by cDNA production using random primers with inclusion of actinomycin D in the first-strand reaction. The ends of the cDNA are repaired, A-tailed and adaptors ligated for indexing (four different barcodes per lane) during the sequencing runs. The cDNA libraries were quantitated using qPCR in a Roche LightCycler 480 with the Kapa Biosystems kit for library quantitation (Kapa Biosystems, Woburn, MA) before cluster generation. Clusters were generated to yield ∼725–825 K clusters per mm^2^. Cluster density and quality were determined during the run after the first base addition parameters were assessed. We ran paired end 2 × 50 bp sequencing runs to align the cDNA sequences to the reference genome.

### Bioinformatic analyses

We obtained 35–55 million of paired 51 bp reads for each sample. The investigator was blinded to sample identities when performing bioinformatic analyses. All the reads were mapped to the human reference genome (GRCh37/hg19) using TopHat (v2.0.13) with a gene transfer file (GTF version GRCh37.70). Low quality mapped reads (MQ>30) were removed from the analysis. The mean insert sizes and the s.d.'s were calculated using Picard-tools (v1.126) (http://broadinstitute.github.io/picard/).

For mRNA-Seq, read count tables were generated using HTSeq (v0.6.0)^53^ and deferential expression (DE) analysis of genes were performed using DESeq (v3.0)^54^. Deferential exon usage was performed using DEXSeq (v3.1)^29^. Two independent biological repeats for control and KD were included in both DEseq and DEXseq. For 293 cells, the mRNA-seq results of the control samples include (1) those from the doxycycline-treated parental Flp-In T-REx 293 cells by us and (2) those from the doxycycline-treated control Flp-In T-REx 293 cells performed by another group unrelated to us (sample GSM1095127 in GSE44976)^31^, and the two AKAP95 KD samples were from the two different clones (miR#8 and miR#12) of Flp-In T-REx 293 cells stably expressing AKAP95 microRNAs after doxycycline treatment. For mouse ES cells, the two biological repeats for both control and shRNA#1-mediated Akap95 KD were independently generated by two different persons in our lab. The read per million normalized BigWig files were generated using BEDTools (v2.17.0)^55^ and bedGraphToBigWig tool (v4). All the downstream statistical analyses and generating plots were performed in R (v3.1.1) (http://www.r-project.org/). Gene ontology analysis was performed at DAVID (http://david.ncifcrf.gov/).

For RIP-Seq, mapped files were passed to Model-based Analysis of ChIP-Seq (MACS) (v1.4.2 20120305)^56^ for peak calling and HOMER (vv3.12, 6-8-2012) (http://homer.salk.edu/homer/)^57^ was used for Motif finding. Bedgraph files generated by MACS were then normalized based on read per million and converted to BigWig files using bedGraphToBigWig. The input RNA-seq files were not subject to MACS. The genomic regions for annotation (intergenic regions, exons, UTRs, distal and proximal introns) were extracted from the GTF file used above. Peak annotations and coverage calculations for these genomic regions were performed using Bedtools and SAMtools (v0.1.18)^58^, respectively. Profile plots were generated by ngs.plot (v2.47)^59^.

### Statistics

All data are shown as mean values with s.d. Unless otherwise specified in figure legends, the unpaired two-tailed Student's *t*-test was used under the assumption of Gaussian (normal) distribution to calculate *P* values and evaluate the statistical significance of the difference between the indicated samples as described in figure legends, and the variances are similar between the groups being statistically compared.

### Data availability

The RIP-seq an RNA-seq data have been deposited in the Gene Expression Omnibus database, with accession code GSE81916. All other data is available from the author upon reasonable request.

## Additional information

**How to cite this article:** Hu, J. *et al*. AKAP95 regulates splicing through scaffolding RNAs and RNA processing factors. *Nat. Commun.* **7,** 13347 doi: 10.1038/ncomms13347 (2016).

**Publisher's note:** Springer Nature remains neutral with regard to jurisdictional claims in published maps and institutional affiliations.

## Supplementary Material

Supplementary Information Supplementary Figures 1-7 and Supplementary Tables 1-3.

Supplementary Data 1 Genes with significantly changed exon usages or intron-bound AKAP95. This dataset has two tabs. One tab lists genes with exon usage significantly affected over 2 fold by AKAP95 KD as well as genes with AKAP95 binding to their intronic regions in human 293 cells. Genes with exon usage downregulated or upregulated over 2 fold by AKAP95 KD are listed separately in indicated columns. The other tab lists genes with exon usage downregulated or upregulated over 1.5 fold by AKAP95 KD in mouse ES cells. All analyses were based on the DEXseq program.
