# Supplementary material for: AKAP95 regulates splicing through scaffolding RNAs and RNA processing factors
Source: Nat Commun. 2016 Nov 8;7:13347. doi: 10.1038/ncomms13347 (PMC5105168; doi:10.1038/ncomms13347)
Supplement: Supplementary Information — Supplementary Figures 1-7 and Supplementary Tables 1-3. [file ncomms13347-s1.pdf]

a

```
  1 MDQGYGGYGA WSAGPANTQG AYGTGVASWQ GYENYNYYGA QNTSVTTGAT YSYGPASWEA
 61 AKANDGGLAA GAPAMHMASY GPEPCTDNSD SLIAKINQRL DMMSKEGGRG GSGGGGEGIQ
121 DRESSFRFQP FESYDSRPCL PEHNPYRPSY SYDYEFDLGS DRNGSFGGQY SECRDPARER
181 GSLDGFMRGR GQGRFQDRSN PGTFMRSDPF VPPAASSEPL STPWNELNYV GGRGLGGPSP
241 SRPPPSLFSQ SMAPDYGVMG MQGAGGYDST MPYGCGRSQP RMRDRDRPKR RGFDRFGPDG
301 TGRKRKQFQL YEEPDTKLAR VDSEGDFSEN DDAAGDFRSG DEEFKGEDEL CDSGRQRGEK
361 EDEDEDVKKR REKQRRRDRT RDRAADRIQF ACSVCKFRSF DDEEIQKHLQ SKFHKETLRF
421 ISTKLPDKTV EFLQEYIVNR NKKIEKRRQE LMEKETAKPK PDPFKGIGQE HFFKKIEAAH
481 CLACDMLIPA QPQLLQRHLH SVDHNHNRRL AAEQFKKTSL HVAKSVLNNR HIVKMLEKYL
541 KGEDPFTSET VDPEMEGDDN LGGEDKKETP EEVAADVLAE VITAAVRAVD GEGAPAPESS
601 GEPAEDEGPT DTAEAGSDPQ AEQLLEEQVP CGTAHEKGVP KARSEAAEAG NGAETMAAEA
661 ESAQTRVAPA PAAADAEVEQ TDAESKDAVP TE
```

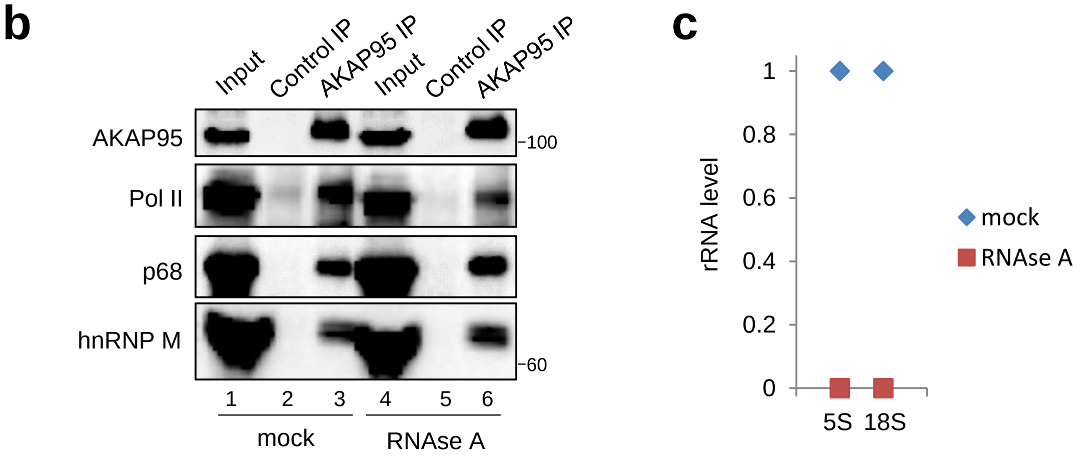

Supplementary Figure 1. AKAP95 associates with proteins involved in transcription and RNA processing through its YG-rich N-terminal region and in an RNA-independent manner.
(a) Human AKAP95 protein sequence. YGG/YG sequences are highlighted in cyan, and RG sequences are in yellow. The cysteines in the two zinc finger domains are in red and underlined.
(b) Immunoblot analysis of endogenous AKAP95-associated proteins following immuno-precipitation by control (normal rabbit IgG) or anti-AKAP95 antibody from mock-treated or RNAse A-treated HeLa cell nuclear extract.
(c) The 5S and 18S rRNA levels in the mock-treated or RNAse A-treated HeLa cell nuclear extract were determined by RT-qPCR.

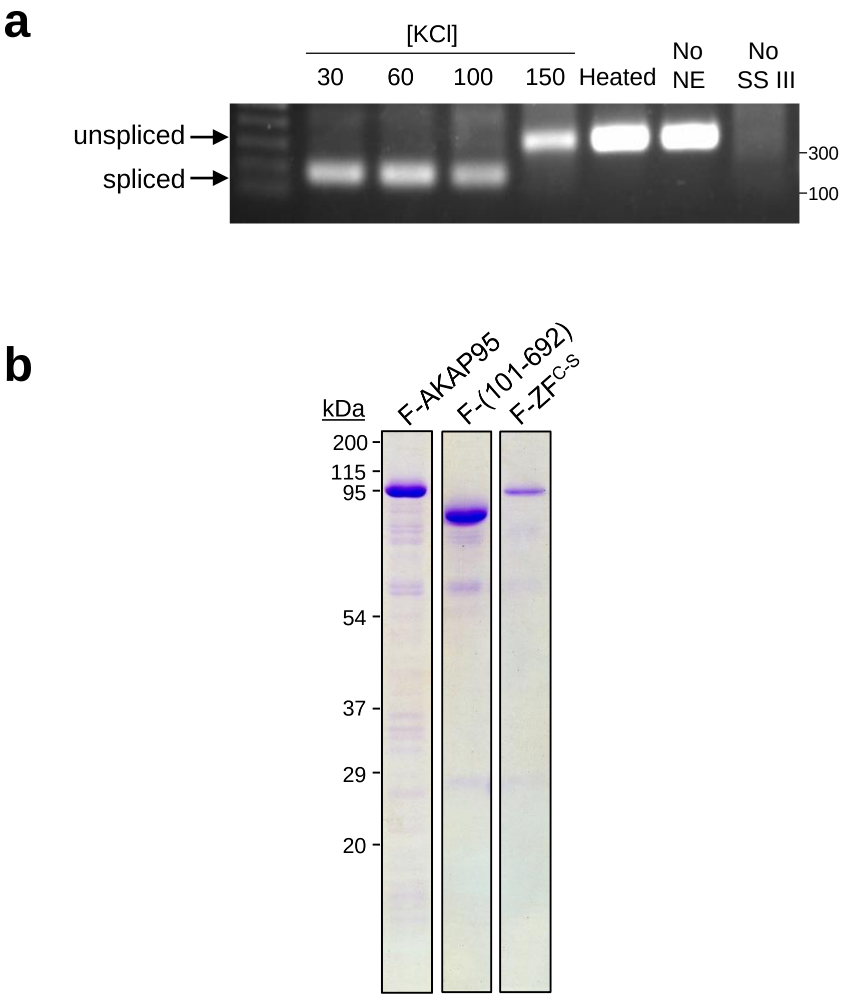

Supplementary Figure 2. AKAP95 is directly required for efficient minigene splicing.
(a) *In vitro* splicing assay under different conditions, including indicated final KCl concentrations in millimolar, using heat-inactivated nuclear extract, or no nuclear extract (no NE). The last lane was from a normal splice reaction but no reverse transcriptase SS III was used in the reverse transcription reaction. All reactions here used HeLa nuclear extract batch 1, which was much more concentrated and thus more active than batch 2 used in Fig. 2e.
(b) Coomassie staining of FLAG-tagged AKAP95 wild type or indicated mutants purified from Sf9 cells.

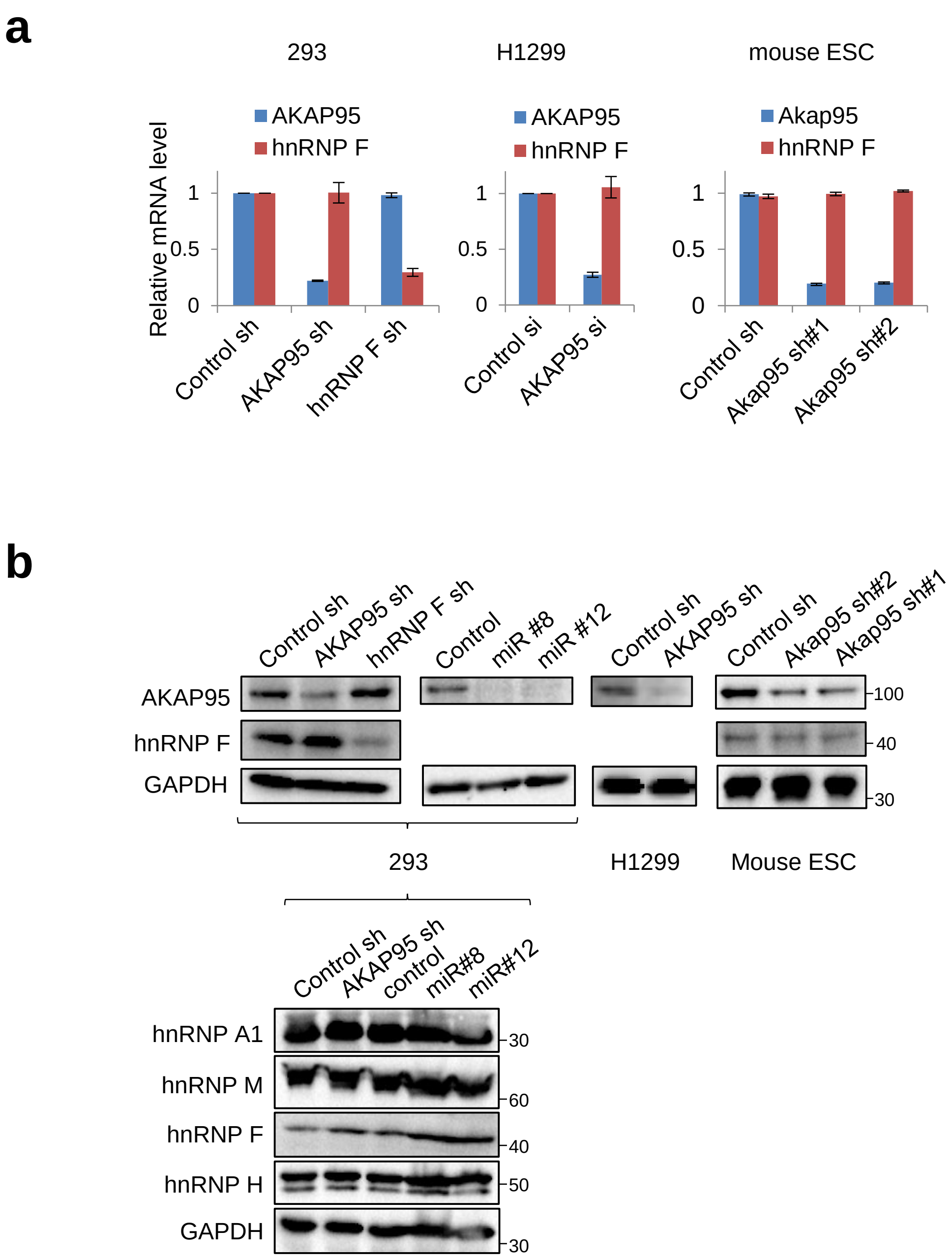

Supplementary Figure 3

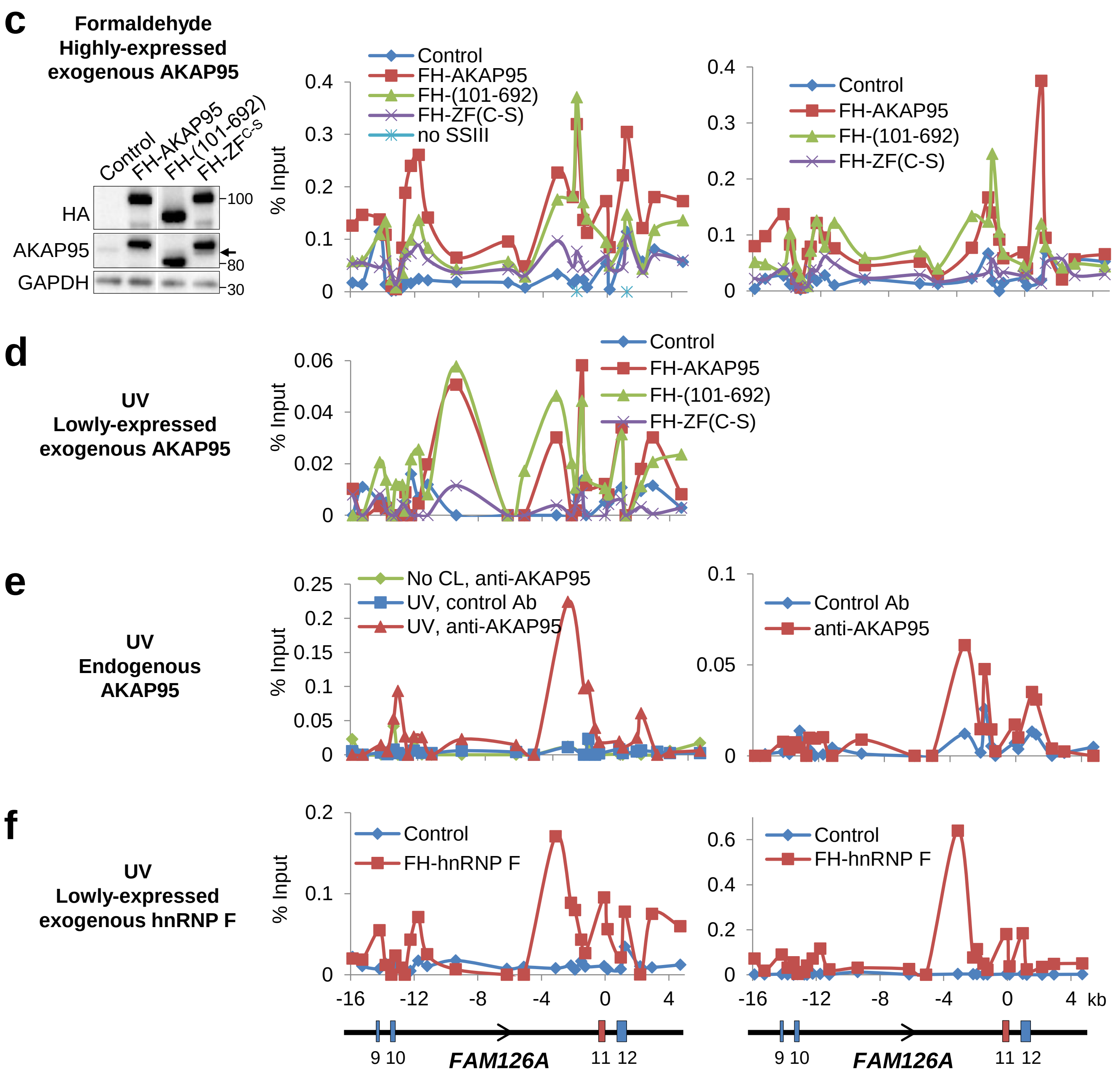

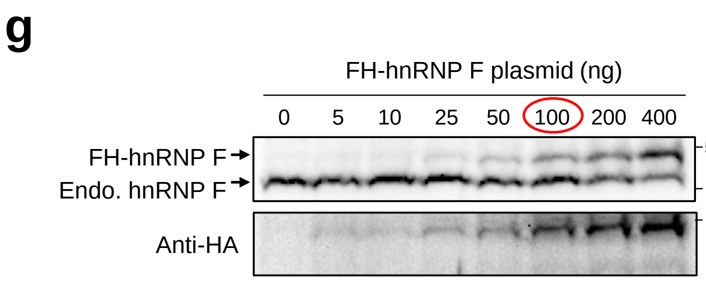

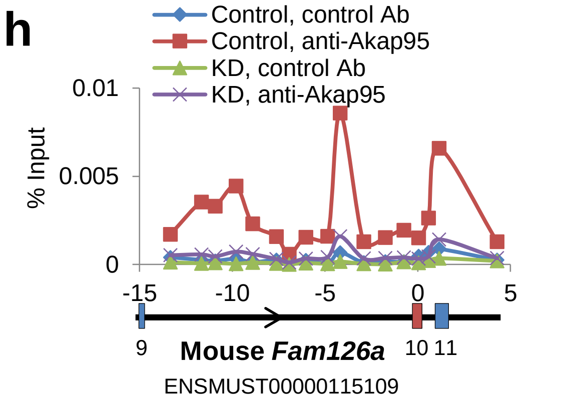

Supplementary Figure 3 (continued)

Supplementary Figure 3. AKAP95 directly regulates alternative splicing of *FAM126A*.
(a) Expression of AKAP95 and hnRNP F was determined by RT-qPCR from the shRNA- or siRNA-treated cells as indicated, and normalized to *GAPDH*. Average ± SD from 3 biological repeats for each are plotted.
(b) Immunoblotting of AKAP95 and hnRNP proteins following AKAP95 KD by shRNA or microRNA (miR#8 and miR#12).
(c) Overexpression of FH-AKAP95 or its indicated mutants was induced by doxycycline in stable Flp-In T-REx 293 cell lines. The arrow points to the endogenous AKAP95 in the western blot on the left. The control is the parental Flp-In T-REx 293 cells. Anti-FLAG RIP assays were performed in these cell lines after formaldehyde-crosslinking, and followed by qPCR by a series of primers at the *FAM126A* locus. Reverse transcriptase was omitted for the “no SSIII” sample. Two independent repeats of RIP assays are shown.
(d) This is another biological repeat of RIP assays shown in Fig. 3b, after UV-crosslinking of cells expressing exogenous wild type or mutant AKAP95 at levels comparable to the endogenous level.
(e) These are two more independent repeats of RIP assays for endogenous AKAP95 shown in Fig. 3c, after UV-crosslinking of normally growing 293 cells.
(f) These are two more independent repeats of RIP assays shown in Fig. 3d, after UV-crosslinking of 293T cells transfected with hnRNP F plasmid dose equivalent to that circled in Supplementary Fig. 3g.
(g) Immunoblot analysis of 293T cell lysates following transfection of indicated amount of hnRNP F plasmid. Anti-AKAP95 and anti-HA antibodies were used for the top and bottom images, respectively. The circled dose represents the plasmid dose used in transfections for RIP assays in Fig. 3d and Supplemental Fig. 3f.
(h) RIP assays using control (normal rabbit IgG) or anti-Akap95 antibody were performed in control or Akap95-depleted (KD) mouse ES cells after UV cross-linking, and followed by qPCR at the mouse *Fam126a* locus.

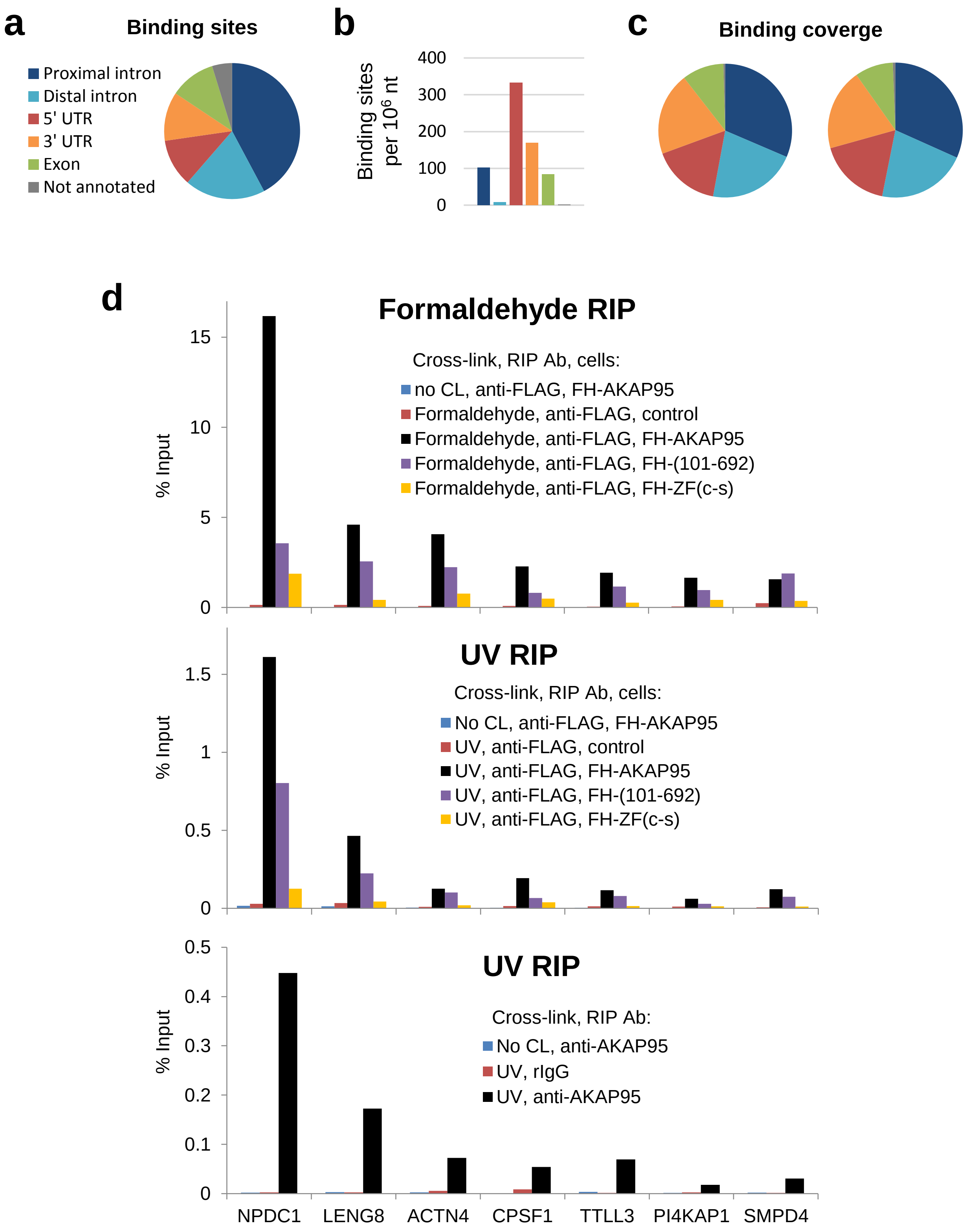

Supplementary Figure 4

e

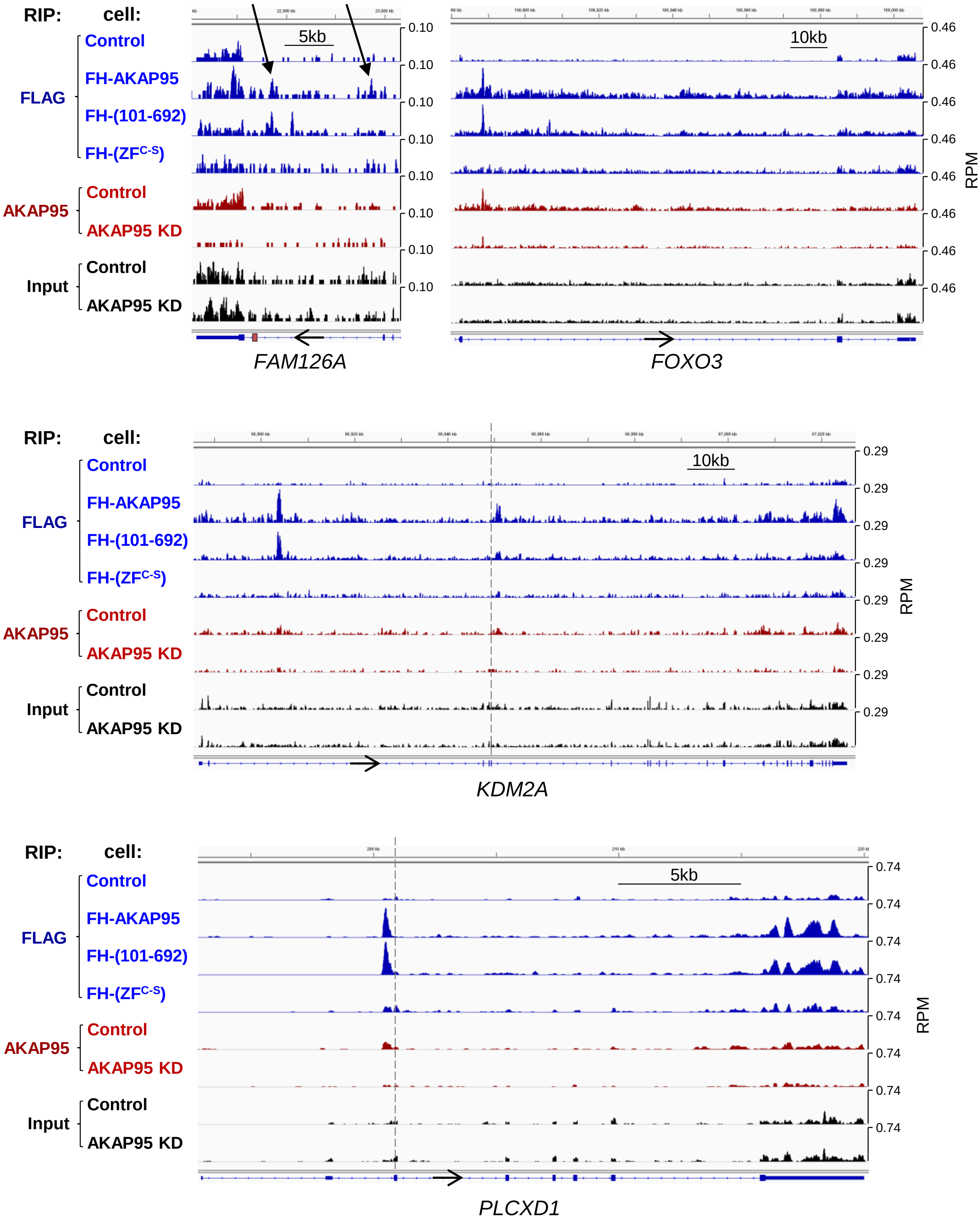

Supplementary Figure 4 (continued)

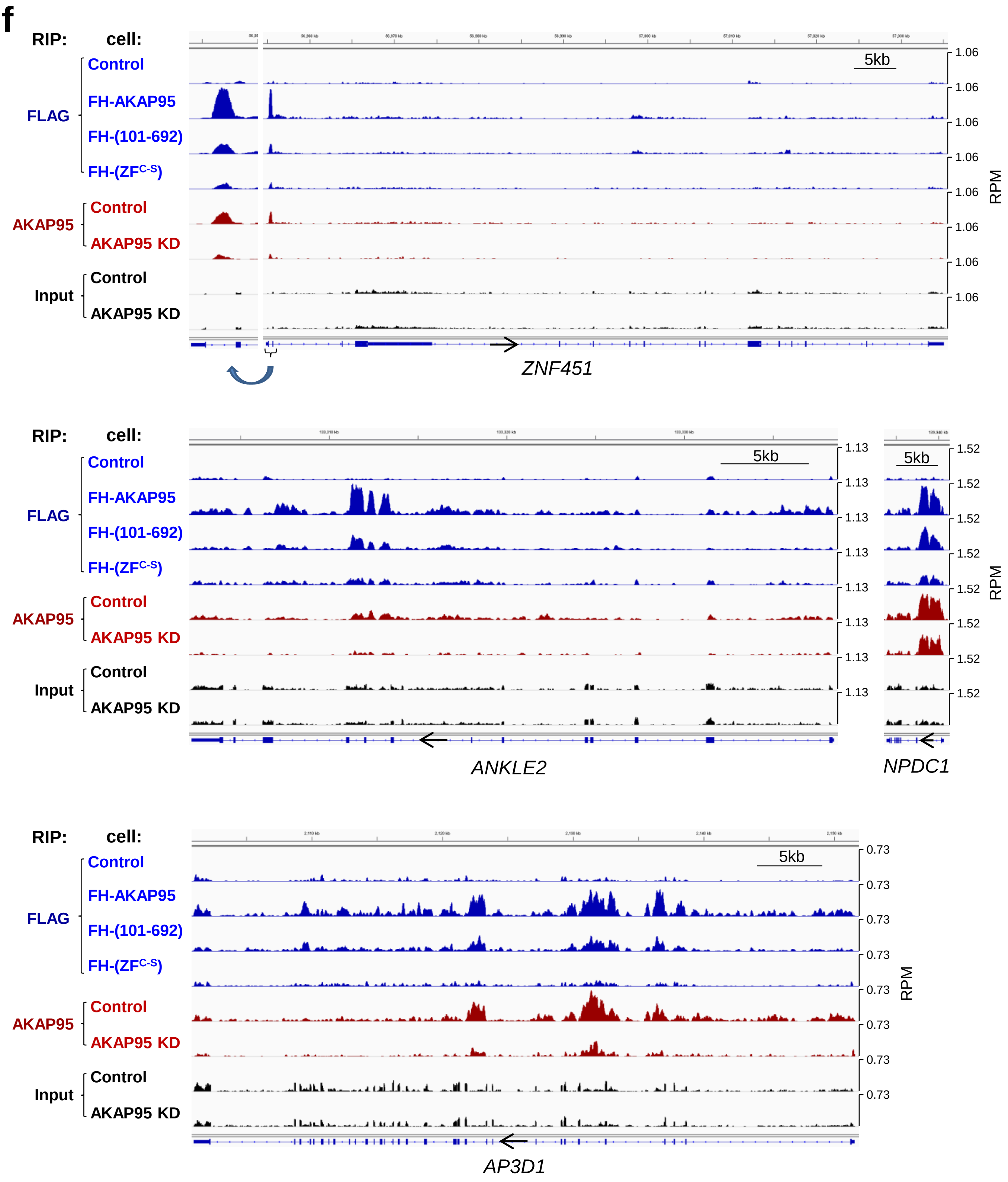

Supplementary Figure 4 (continued)

Supplementary Figure 4. AKAP95 preferentially binds to introns of cellular pre-mRNAs.
(a) Distribution of the numbers of FH-AKAP95 binding sites within different categories of genic regions, as calculated from MACS peak counts of the anti-FLAG RIP results from 293 cells induced to express FH-AKAP95. The color legend applies to a-c.
(b) FH-AKAP95 binding sites in each 1 million nucleotides of different genic regions, using anti-FLAG RIP results from 293 cells induced to express FH-AKAP95.
(c) Distribution of the genomic region coverage of AKAP95 binding sites within different categories of genic regions, as calculated from MACS peak coverage of anti-AKAP95 RIP results from 293 cells (left) or anti-FLAG RIP results from 293 cells induced to express FH-AKAP95 (right).
(d) qPCR results of UV RIP assays for AKAP95 binding at introns of indicated genes, where strong binding peaks were found in the formaldehyde-based AKAP95 RIP-seq.
(e and f) Representative RIP profiles. In blue are the Anti-FLAG RIP-seq profiles from parental 293 cells (control) or stable 293 cells overexpressing the FLAG-tagged wild type AKAP95 or indicated AKAP95 mutants. In red are the anti-AKAP95 RIP-seq profiles from parental 293 cells (control) or 293 cells stably expressing AKAP95 microRNAs (AKAP95 KD). In black are the sequencing profiles of total input RNAs from the corresponding cells for the anti-AKAP95 RIP-seq. All profiles have the same y-axis scale for each gene so that they are directly comparable. Note that, compared to the FH-AKAP95 binding, FH-ZFc-s binding was drastically reduced at all shown transcripts, but FH-(101-692) binding was either unaffected **(e)** or modestly reduced **(f)** at different transcripts or different regions within the same transcript. Arrows at the FAM126A panel point to the two intronic binding peaks shown by qPCR in Figure 3. The broken vertical lines in the KDM2A and PLCXD1 panels were positioned at an exon to clearly show that the nearby AKAP95 binding peaks were not at the exon, but rather, proximal to the exon. In the ZNF451 panel, the left sub-panel shows a zoomed-in image of the first intron. Arrows on the gene diagrams indicate the direction of the gene transcription.

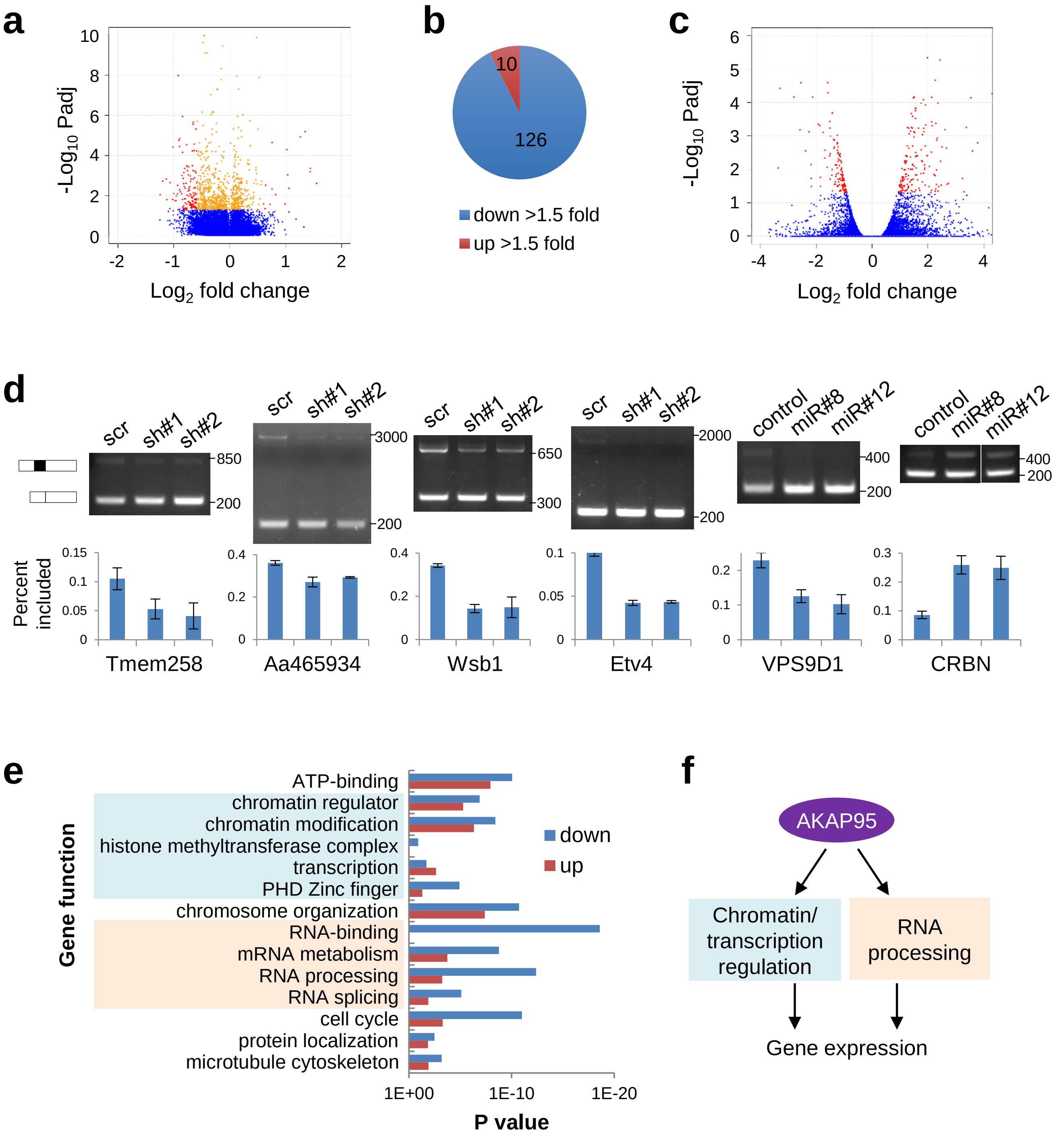

Supplementary Figure 5. AKAP95 preferentially promotes exon inclusion.
(a) Volcano plot for exons whose normalized usage was significantly affected (Padj<0.05 for red and yellow, red for fold change over 1.5) or not (Padj>0.05, blue) by Akap95 shRNA #1 compared to scramble shRNA in mouse ES cells. Fold change is the ratio of the normalized exon level in the AKAP95 knockdown over that in the control cells. See Supplementary Table 2 for the gene list.

(b) Pie chart showing the number of exons whose normalized usage was significantly (P<0.05) reduced (blue) or enhanced (red) by Akap95 shRNA #1 compared to scramble shRNA in mouse ES cells.
(c) Volcano plot for genes whose expression was significantly affected (Padj<0.05 for red and yellow, red for fold change over 1.5, and only very few significantly affected genes were changed less than 1.5 fold) or not (Padj>0.05, blue) by Akap95 shRNA #1 compared to scramble shRNA in mouse ES cells. Fold change is the ratio of the normalized exon level in the AKAP95 knockdown over that in the control cells.
(d) Validation of the DEXseq analyses on the effects of AKAP95 knockdown on inclusion of exons in mouse ES cells (for Tmem258, Chpt1, As465934, Wsb1, and Etv4) or human 293 cells (for VPS9D1 and CRBN). Following quantification of band intensity by the ImageJ program, the percentage of the PCR product representing exon 11-included transcript in the total transcripts was calculated and shown as "percent included" on the Y axis. Because the exons shown in this panel appear to be mostly skipped, plotting "percent skipped" as in Fig. 3a shows only modest (yet statistically significant) changes (data not shown). Average ± SD from biological duplicates are plotted. P<0.05 between control and KD for all these genes except *Tmem258.*
(e) Gene ontology analyses of genes that contain significantly (P<0.1) affected exons upon AKAP95 knockdown by shRNA #1 in mouse ES cells, based on 632 DAVID IDs with down-regulated exons and 322 DAVID IDs with up-regulated exons. Gene functions in light blue shade are for chromatin/transcription regulation, and gene functions in pink shade are for RNA binding and processing.
(f) A diagram showing that AKAP95 may regulate gene expression through regulating alternative splicing of RNAs encoding factors involved in chromatin and transcription regulation and factors involved in RNA processing, based on results in Fig. 5f and Supplementary Fig. 5e.

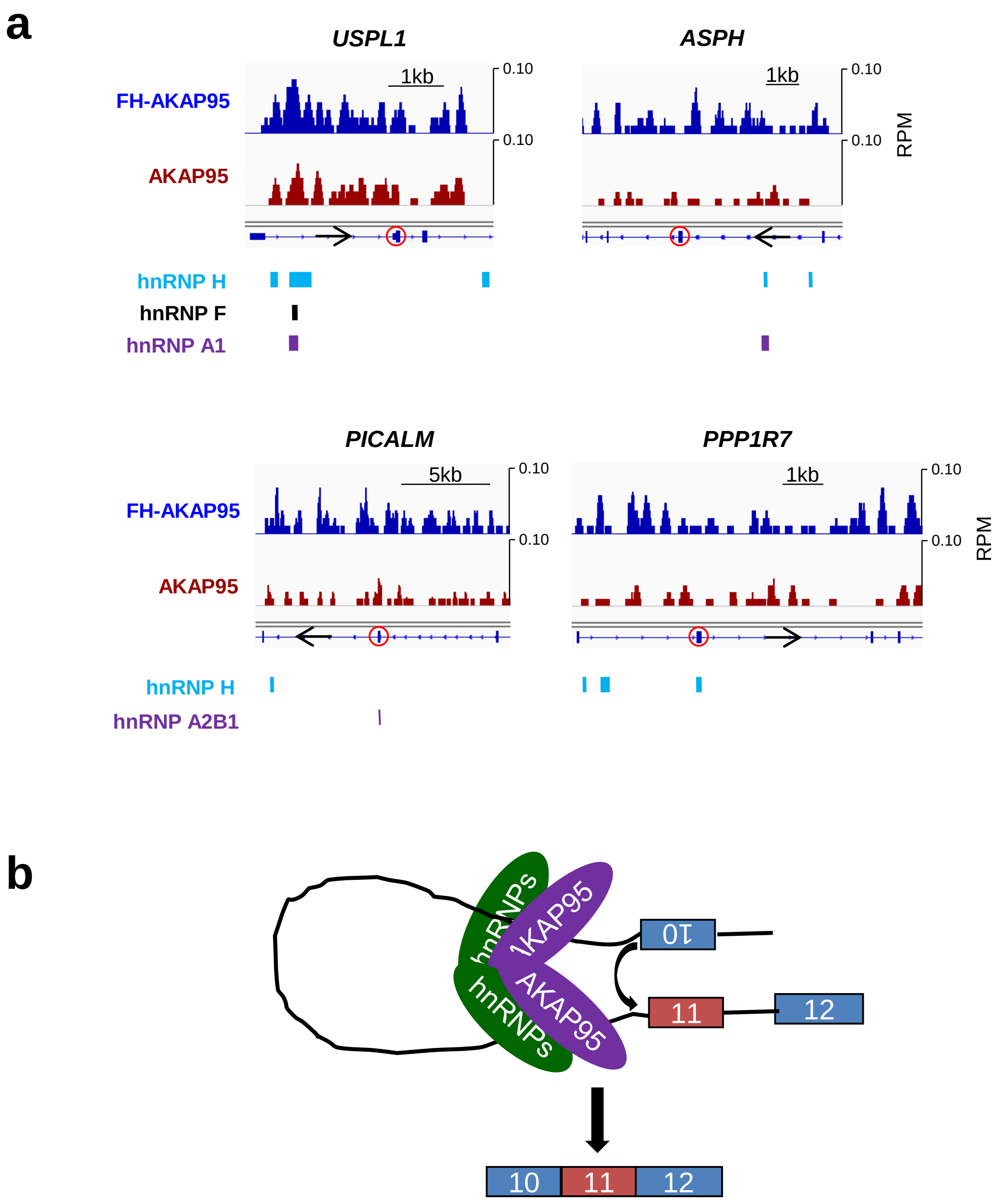

Supplementary Figure 6. A possible model for the role of AKAP95 in splice regulation.
(a) RNA binding profiles of exogenous and endogenous AKAP95 (blue and red, respectively) at RNA regions flanking the exons (marked by red circle) regulated by AKAP95 and hnRNPs, as shown in Fig. 6a. Shown at the bottom are binding regions of hnRNP proteins taken from http://rnabind.ucsd.edu/.
(b) The model shows how AKAP95 might regulate the splicing of *FAM126A* pre-mRNA by binding to intronic regions near the splice sites and also facilitating splice site communication via its interaction with hnRNPs and/or itself.

For Fig. 1d

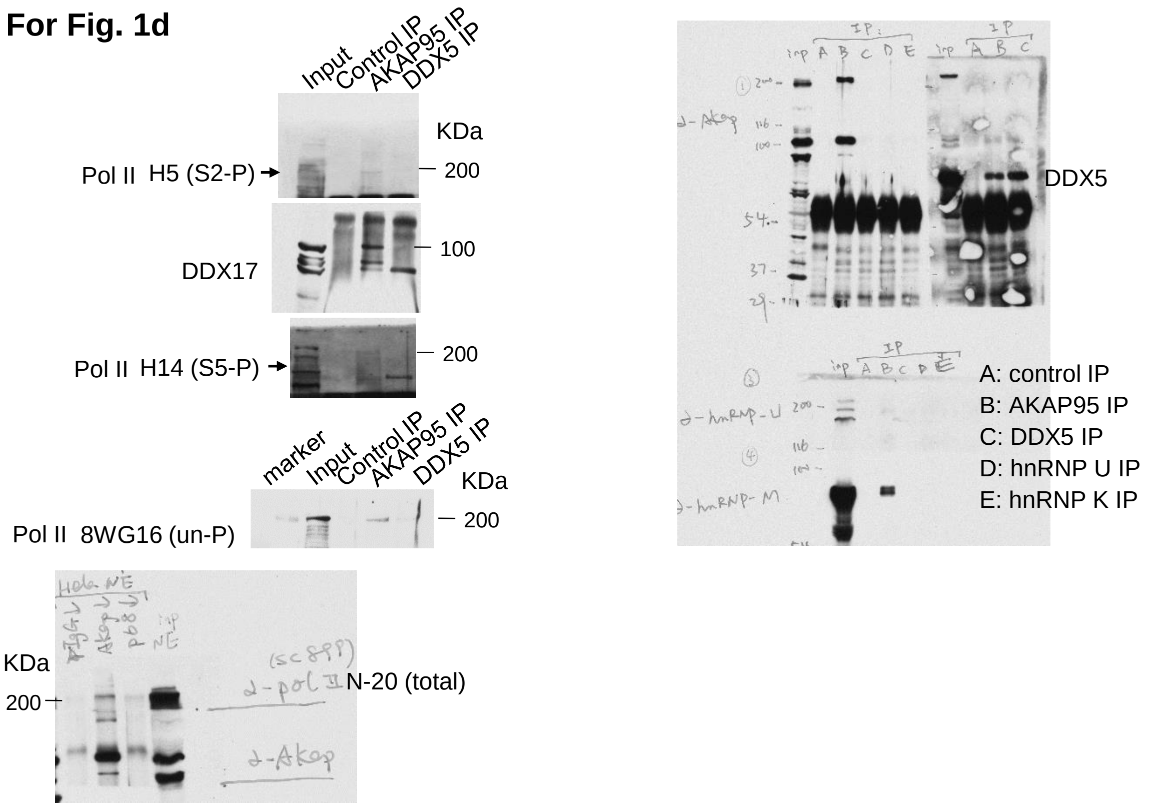

For Fig. 1e

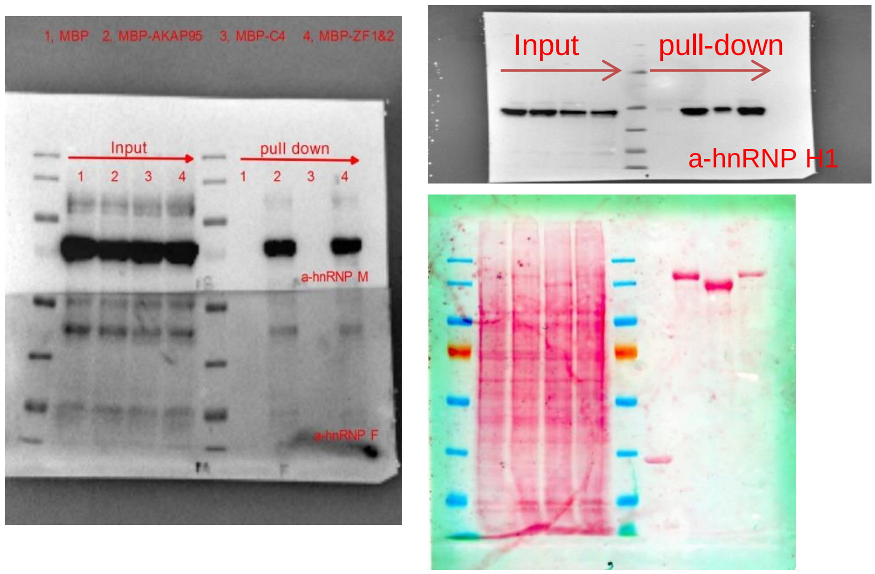

Supplementary Figure 7

For Fig. 1f

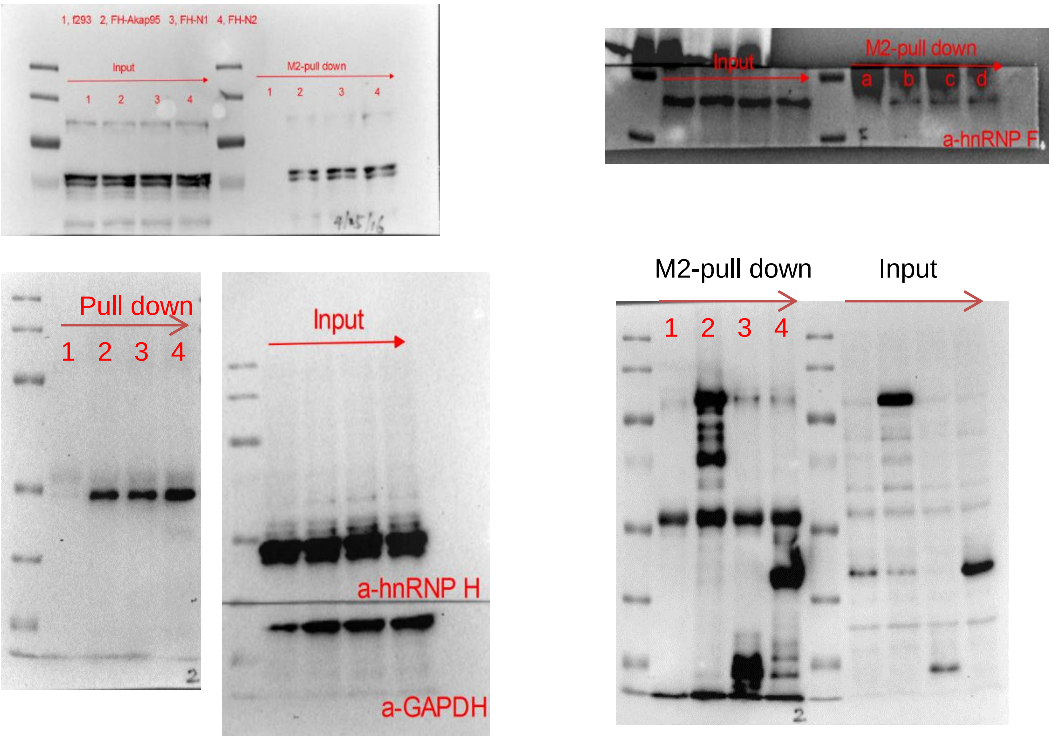

For Fig. 1g

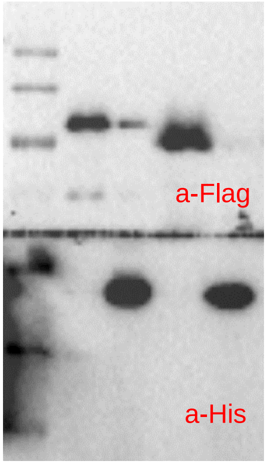

For Fig. 1h

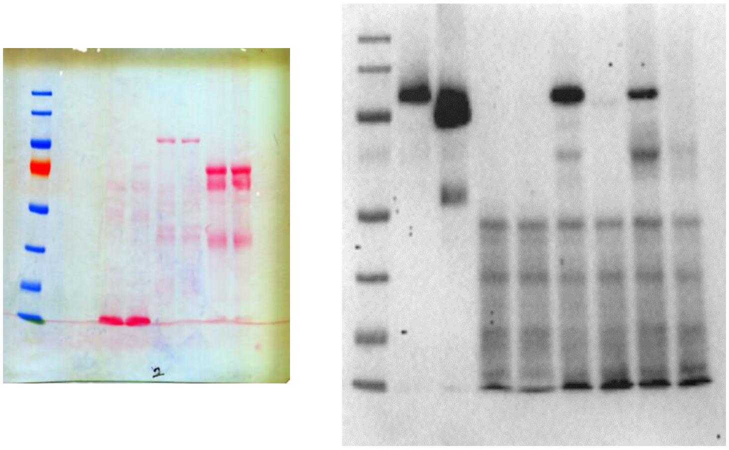

Supplementary Figure 7 (continued)

For Fig. 2c

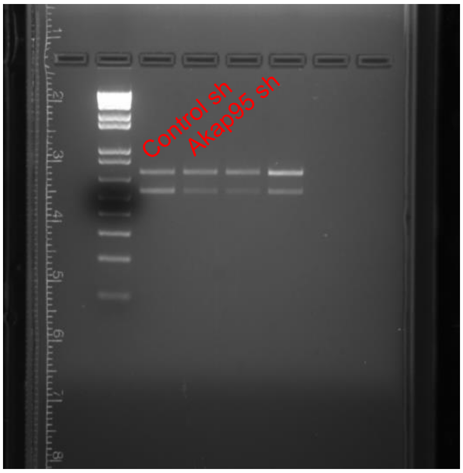

For Fig. 2d

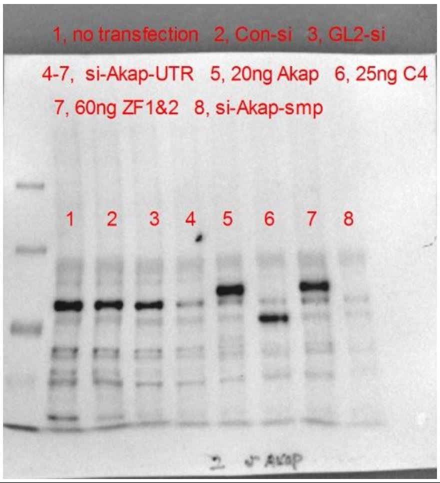

For Fig. 2e

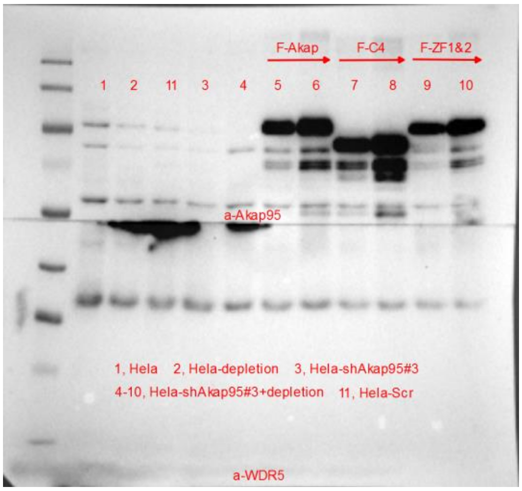

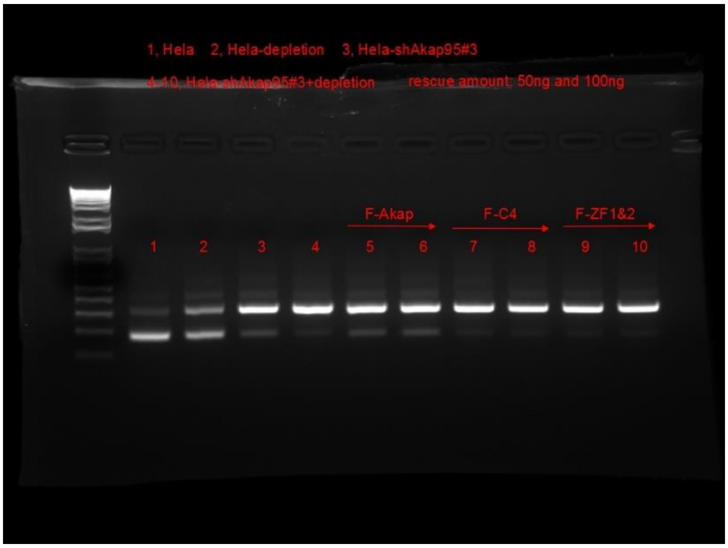

For Fig. 3a

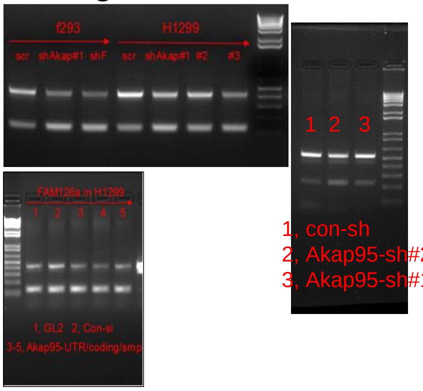

For Fig. 3b

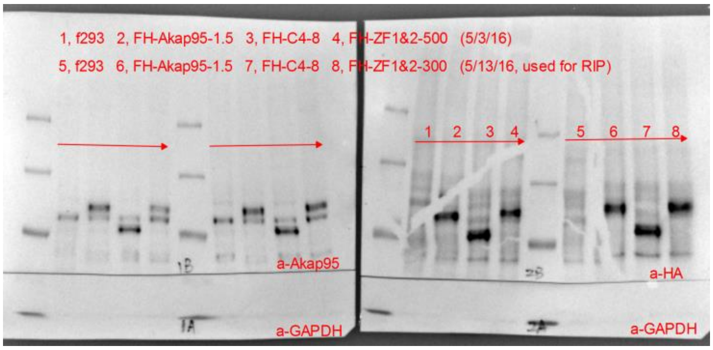

Supplementary Figure 7 (continued)

For Fig. 6a

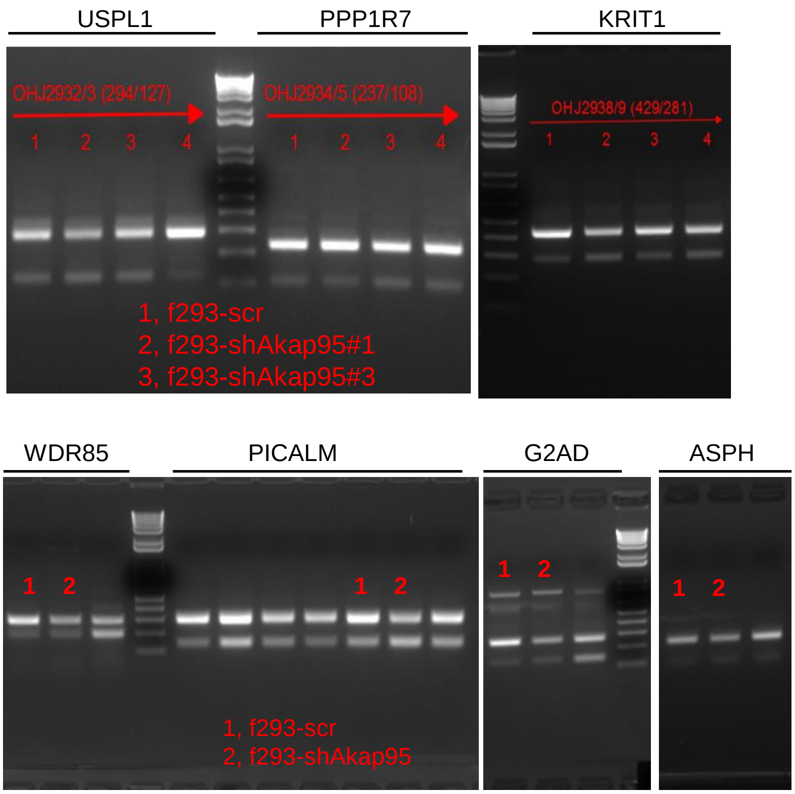

For Fig. 7a

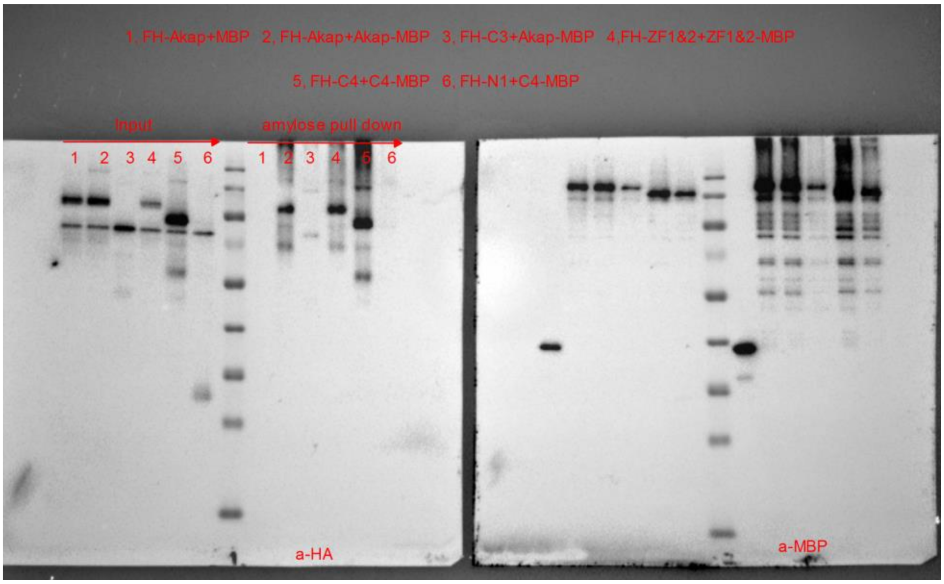

Supplementary Figure 7. Uncropped blots. Note that some of these blots are merged with marker to show size, and blots in the main figures are unmerged and some may be from different exposure times.

Supplementary Table 1. Mass spectrometry results for AKAP95-associated proteins. Major proteins co-immunoprecipitated with FH-AKAP95 or FH-AKAP95 (101-692) were identified by MALDI mass spectrometry, categorized and listed in the left column, and followed by their molecular weights (MW), mass spectrometry protein scores and numbers of matched peptides. Mass spectrometry protein scores are defined as the combined ion scores of all peptides matched from mass spectrometry peak lists. The ion score is calculated by the Mascot software as -10Log P, where P is the probability that the observed match of each peptide sequence is a random event. As the same protein was often detected in multiple segments excised from the gel (Figure 1b) with different scores and numbers of matched peptides, only the highest score and number of matched peptides for that protein are listed. Proteins in red are those with protein scores over 200. NF, not found.

		FH-AKAP95		FH-(101-692)	
	**MW (kD)**	**score**	**# peptides**	**score**	**# peptides**
**hnRNPs**					
hnRNP M	78	3690	198	58	7
hnRNP H1	49	1618	86	184	14
hnRNP H2	49	1207	71	156	14
hnRNP H3	37	187	13	84	4
hnRNP F	46	1107	69	53	9
hnRNP D	32	275	21	NF	
hnRNP U	90	252	34	NF	
hnRNP K	51	371	29	NF	
**DEAD-box helicases**					
DDX5 (p68)	67	756	62	NF	
DDX17 (p72)	73	920	59	NF	
DDX17 (p82)	82	789	55	NF	
DDX3X	74	196	16	NF	
DDX3Y	74	130	13	NF	
RNA helicase A (DHX9)	142	98	23	NF	
**Other RNA-associated proteins**					
RBM14 (CoAA)	70	305	26	NF	
EWS	69	58	11	NF	
NHN1 (ZC3H18)	107	168	9	NF	
HA95	73	157	8	NF	
ZNF326	66	168	10	NF	
DBC1 (CCAR2)	103	300	13	NF	
FAM120A (C9orf10)	118	110	7	NF	
KHDRBS1 (SAM68)	48	77	21	NF	
ZCCHC8	54	44	3	NF	

NOP56	66	267	16	NF	
NOL9	80	83	6	NF	
NOP58	60	44	7	NF	
SFRS2	166	46	8	NF	
SFRS14 (SUGP2)	111	65	10	NF	
**Others**					
KLHL15	71	110	13	NF	
PELP1	138	54	9	NF	
DNA-PKc	470	49	10	NF	
CAD	245	55	7	NF	
NFYC	51	42	4	NF	

Supplementary Table 2. shRNA, siRNA, and miRNA sequences

RNA format	Species	target	Sequence 5'-3'
shRNA	Mouse	Akap95 #1	GCCCGACAAGACAGTAGAATT
		Akap95 #2	GCCTGTTCTGTATGCAAGTTT
	Human	AKAP95	ATTAGTTACTACCACTCAAAT
siRNA	Human	AKAP95 3' UTR	GAAAGGAGGCUGUAGAAUA
		AKAP95 coding	GAAUUGAUGGAGAAAGAAA
		AKAP95 ON-TARGET plus SMARTpool	GUCCAUGGCUCCCGACUAC
			GGAACGAGCUGAACUACGU
			GAACAACAGACAUAUAGUG
			GAUCGAGGCUGCUCACUGC
MiRNA	Human	AKAP95 miR 1	TAAAGTTGGAACTGCTTCCGT
		AKAP95 miR 2	TTTCGGAGAAATCTCCTTCAC

Supplementary Table 3. PCR or qPCR Primers

**App.**	**Species**	**gene**		**Forward primer (5'-3')**		**Backward primer (5'-3')**
expression	Human	GAPDH		CCTTCATTGACCTCAACTACATGG		TCGCTCCTGGAAGATGGTGATGGG
expression	human	ACTB		CCTTCAACACCCCAGCCATGTACG		GGCACAGTGTGGGTGACCCCGTC
expression	human	5S rRNA		GATCTCGTCTGATCTCGGAAG		GGTATTCCCAGGCGGTCT
expression	human	18S rRNA		ACAGGATTGACAGATTGA		TATCGGAATTAACCAGACA
expression	human	AKAP95		GCCAAGGCCAATGATGGCGGCCTG		GCCCCTGCCTCCTTCCTTGGAC
expression	human	hnRNP F		GGGAAACACAAGGAGAGGATAG		GCACGGACATGAACTTCAGA
expression	mouse	Gapdh		CATCTTCTTGTGCAGTGCCAG		GGCAACAATCTCCACTTTGCC
expression	mouse	Akap95		TGTACCGACAACTCAGACTCG		GTAGGACTCGTATGGCTGGAA
Splicing-PCR	pTN23/24	minigene		CGCGGATCCGTACTCCCTCTCAAAAGC		CCGGAATTCCTTCTCCGCCTGAGCCTC
Splicing-qPCR	pTN24	Spliced minigene		TTCACCAGCTGTTGGGTAA		TGAGGTTGTTGGTGACATTCT
splicing	mouse	Fam126a		CACACCAACCTCCTCTAGAATATC		CGTCTCTGGTTCTTTCCTACAC
splicing	human	FAM126A		TGCAGTAACCAGCATGTCAA		CTAGACGCAGCCCTGGAATA
splicing	human	KRIT1		ATCTCGGTGGTCCAACTCAG		TGGCAGTATTCTTTGGACGA
splicing	human	PPP1R7		AGTCGCAGGAGATGATGGAG		TCCCTATGCGATAGTGATTCAA
splicing	human	ASPH		TCGAAGATGAAGCAAAAGAACA		CTTCCACGTGGTAACTATGCTC
splicing	human	WDR85		CTGGTCGAGGTCCAAAGAAA		TCCTCCCCAACCCTCTCTAT

splicing	human	USPL1		ACGTTGCAACTAGGGTGGAG		CAAGCAGGGCAATACTCATCT
splicing	human	G2AD		AGCTGGCCAAACTGCTCTAC		CAGGTCTCGGCACATCTCA
splicing	human	PICALM		GCCCAATGATCTGCTTGATT		ATTAAGGCCAGCTGAAGGGT
splicing	mouse	Tmem258		GAGCTCGAAGCCATGAGTAG		GACTCCAAAGCCCATGAAGA
splicing	mouse	Aa465934		TTTGAGAACCTGTGTGTAAGTCC		ATTGATTCCAATTCAGGGCAGA
splicing	mouse	Wsb1		CATTCTCTCCCGACTGTTCTATG		TACTCAGGAGTCTGGACATTACT
splicing	mouse	Etv4		GCAGAACTTCAAGCAGGAGTA		GGGAATGGTCGAAGGGATTT
splicing	human	VPS9D1		GCCATGAAGCTTGCCAAC		GGCCCTCTCCAGACACT
splicing	human	CRBN		GGAAGCACAGTTTGGAACAAC		CTGGCACTTATTGAGGGATTCT
**App.**	**Species**	**gene**	**Forward start***	**Forward primer (5'-3')**	**Backward start***	**Backward primer (5'-3')**
RIP	human	FAM126A	-15948	GGCAGAAGTGACCTAGGATTG	-15806	GAGGACGAAGCAGGAGTATTAAG
RIP	human	FAM126A	-15319	CCTATACCATCAAGGGCAGTTT	-15204	CATACAAGTGGGAGCCTGTATC
RIP	human	FAM126A	-14216	GGAGAATGGGATCTAGCTCAA	-14136	CAGCAATGGCTCTGGATATAAT
RIP	human	FAM126A	-13849	CTCCCGAGTAGCTGGGATTA	-13799	GATCAGCCTGACCAACATGAA
RIP	human	FAM126A	-13487	TCCCTCTTAGCCTCCTATTCTT	-13374	CAACATGAGCAGATAGAGCAGATA
RIP	human	FAM126A	-13232	ACACCAACTTCCTCTCGAATATC	-13121	ACAATGGTTTGACTTGAAGAGTTAC
RIP	human	FAM126A	-12811	TCAGTGAACTCTTAAGCAAGTGA	-12705	CGTCAACACTATGGCATTCTTTAC
RIP	human	FAM126A	-12633	TCCCTTCTTGTCTGCTTCATT	-12537	AAGGGAGGGAAATGGGAAAG
RIP	human	FAM126A	-12279	GTACTGGTTGTGTTGTGGTTTC	-12174	CAATGGCAACTGTTCTCATCAC
RIP	human	FAM126A	-11800	ACTGCTACAGGAAGCACAAG	-11695	CTGCAGGACCTACATTTCTCTC
RIP	human	FAM126A	-11232	TCCCTTTATCCAGTGGACTCTA	-11133	GGAAAGCCAAGACTACCCTAAA
RIP	human	FAM126A	-9444	TTGGAGAGATCTGTTTGACAACT	-9345	CACATTAAGTTAAGCCATGTCCATAC
RIP	human	FAM126A	-6223	ACTCCTCCTTTCTCCACCTT	-6121	ACAGGTCAGCAACACCTATTAC
RIP	human	FAM126A	-5180	TCCCAGACCACCCATCTAA	-5044	GGAACAGAGCTCACACTGTAATA
RIP	human	FAM126A	-3179	AAGGAAGAGGGTATGTGAAACG	-3048	CTTCTCCTGGTGGCTGAATG
RIP	human	FAM126A	-2222	GTTGTGTAGCTCTTCCTGAACT	-2080	TTGCCCTAAGTCGCTTTCTT
RIP	human	FAM126A	-1960	TGGACTGGAGATCCCTTTAGTA	-1844	GAGGTCACTGCAACTGAGATAA
RIP	human	FAM126A	-1558	GGCTCCCAGAAGTGCATATT	-1446	CTGGGAACCACTGCTCTATTC
RIP	human	FAM126A	-1305	CAGCTAAGTTAGGGAACCACAG	-1207	CAGACTCCCACTAATGATGCTAAA
RIP	human	FAM126A	-119	GAATTGGGCATCCTTGTCATTC	-15	CTGAGCATCAATCTCTCCTAGTG
RIP	human	FAM126A	81	TGGAGGATGGAATAAACTCACC	198	CTGTGCAACTGCTGCTTAAC
RIP	human	FAM126A	936	GGAAATACAGAATTAACAGGTCAAGAAG	1015	GACGCAGCCCTGGAATAAA
RIP	human	FAM126A	1177	CAGAGAGCCCAAAGTGAGAAT	1278	CTTAGCCAAACCATGGCTACTA
RIP	human	FAM126A	2132	TGGTAACCCAGTGCCTAGAA	2189	ACAACACACCTACCAGCAATC
RIP	human	FAM126A	2889	GACCGTTGAGGAAGACCTAAG	2938	GTGACTACAGCAAGGGACTATG
RIP	human	FAM126A	4666	AAATGTCTTCCCTTCCCTTCC	4713	AGTCTTGAGAGCTTGCCTTAAA
RIP	mouse	Fam126a	-13329	AGTAAGGAAGGTACAAGGTGTATTC	-13238	CCTCGTATTGACATGCTGGTAA
RIP	mouse	Fam126a	-11662	GACTGTCTCAGGCTACAACTTC	-11564	ATCATAAACAGGAGGCACTCAA
RIP	mouse	Fam126a	-10915	CCACAGTGGCCATTCTGTATT	-10805	CTTACCCAGGATCCCATCAAAC
RIP	mouse	Fam126a	-9801	GCTCGCTCACCTTCTTCTTT	-9693	CCTCTGATGGATGCTTGTCTTC
RIP	mouse	Fam126a	-8905	GAGTGTCTGTGTCTGCTCTTAAA	-8777	AACGACTGGCTTGGAAAGTTA
RIP	mouse	Fam126a	-7623	CTGGCAAGTGGTTGCAAATAA	-7510	GGACAGACACACAGAGGAAAG
RIP	mouse	Fam126a	-6922	TTTCAGAGGTGGGAGGGATTA	-6823	GTTGGTACCTAAGAGGCTGAAG
RIP	mouse	Fam126a	-6022	GGAAGGTCTGGAGTCTGTTATG	-5936	ATGGATGTACTGAGCACCTATG
RIP	mouse	Fam126a	-4849	AGGATGTGTATGGCTCTTTGG	-4732	AGGCTCCTGAAACTTCTTTGT
RIP	mouse	Fam126a	-4166	GTGGAGGTCAGAGGACAATTT	-4069	TAGGGAAAGGCACTTGCTATG
RIP	mouse	Fam126a	-2896	GCATATGAAATGTAGGGATGTTGAG	-2802	AGCTGGAAGGCACATCTTAC
RIP	mouse	Fam126a	-1729	TTCTTGACGTGGTTTCTACAGTAT	-1630	GGTGTGTGGCAGAGGTTATAG
RIP	mouse	Fam126a	-745	GTCTCAATAGGAACCTGAAGAAATG	-624	CAATCATGCCTTGGTGCTTAG
RIP	mouse	Fam126a	-26	GACATTGACGCCCAGCATA	99	GGACATTCCATCCTCCATCATT
RIP	mouse	Fam126a	515	TGGAGAACACAACCTTCCTAAA	651	TGCAAAGAGCAGAAGCAATTAG
RIP	mouse	Fam126a	1103	GAGCGAAGGAGAGAGAAACTG	1206	CTTCAGAGAGAGAAGCTCAAGG
RIP	mouse	Fam126a	4219	CCAAGTGAAGAGAGAAGCAGAG	4308	GTGCTACAGAGATGGGCTTAAA
RIP	human	NPDC1	139938239	CCCTAGTTCCTAACCACACTTTC	139938338	TCTCCAGCACCAGGATAAGA
RIP	human	LENG8	54970232	CAGTGTCCTTCGTTCACTAGAC	54970334	CACCTCAATCACCAGACAGAA
RIP	human	ACTN4	39200494	GAGTATGGAGCCCGACAAAG	39200592	AGTGCATGGCTAGGAAATGG
RIP	human	CPSF1	145621175	GGTGGTGTGGTGGCTTAAATA	145621250	CTTGGCTGCTCTGTGACTTT
RIP	human	TTLL3	9859493	GCTGAAGCTCTGGCTCATATT	9859606	GGGCACATTAGGGAAGGATAAG
RIP	human	PI4KAP1	20386310	GGGAAGGTGGTTGGAATTAGAT	20386399	CTGACCTACACCTGTCCTTTG
RIP	human	SMPD4	130914495	CCATACAAACAGCCCACTCT	130914597	CTGCCATCTCTTGCATCTCATA

* The position is relative to the center of exon 11 of human *FAM126A* or exon 10 of mouse *Fam126a*, which is set as "0". For the rest genes in RIP assays, the position is its chromosomal coordinate on hg19 (GRC37).
